# Prioritized Na^+^ Adsorption-Driven Cationic Electrostatic Repulsion Enables Highly Reversible Zinc Anodes at Low Temperatures

**DOI:** 10.1007/s40820-025-01889-9

**Published:** 2025-09-01

**Authors:** Guanchong Mao, Pan Xu, Xin Liu, Xingyu Zhao, Zexiang Shen, Dongliang Chao, Minghua Chen

**Affiliations:** 1 Key Laboratory of Engineering Dielectric and Applications (Ministry of Education), School of Electrical and Electronic Engineering, Harbin University of Science and Technology, Harbin, 150080 People’s Republic of China; 2 Laboratory of Advanced Materials, Aqueous Battery Center, Shanghai Key Laboratory of Molecular Catalysis and Innovative Materials, Electron Microscope Center of Fudan University, Shanghai Wusong Laboratory of Materials Science, and Faculty of Chemistry and Materials, Fudan University, Shanghai, 200433 People’s Republic of China

**Keywords:** Low-temperature resistant, Organic-free additive, Aqueous batteries, High stability

## Abstract

Aqueous zinc metal batteries (AZMBs) are promising candidates for renewable energy storage, yet their practical deployment in subzero environments remains challenging due to electrolyte freezing and dendritic growth. Although organic additives can enhance the antifreeze properties of electrolytes, their weak polarity diminishes ionic conductivity, and their flammability poses safety concerns, undermining the inherent advantages of aqueous systems. Herein, we present a cost-effective and highly stable Na_2_SO_4_ additive introduced into a Zn(ClO_4_)_2_-based electrolyte to create an organic-free antifreeze electrolyte. Through Raman spectroscopy, in situ optical microscopy, density functional theory computations, and molecular dynamics simulations, we demonstrate that Na^+^ ions improve low-temperature electrolyte performance and mitigate dendrite formation by regulating uniform Zn^2+^ deposition through preferential adsorption and electrostatic interactions. As a result, the Zn||Zn cells using this electrolyte achieve a remarkable cycling life of 360 h at − 40 °C with 61% depth of discharge, and the Zn||PANI cells retained an ultrahigh capacity retention of 91% even after 8000 charge/discharge cycles at − 40 °C. This work proposes a cost-effective and practical approach for enhancing the long-term operational stability of AZMBs in low-temperature environments.

**Supplementary Information:**

The online version contains supplementary material available at 10.1007/s40820-025-01889-9.

## Highlights

- The introduction of low-cost, low-reduction-potential Na^+^ into aqueous Zn-based battery electrolytes suppresses Zn^2+^ aggregation at the anode interface through preferential Na^+^ adsorption and inter-cationic electrostatic repulsion, thereby enabling homogeneous Zn deposition and significantly enhanced low-temperature reversibility of Zn anodes.
- Na^+^ with low ionic potential spontaneously adsorbs at the anode–electrolyte interface, effectively reducing solvated water molecules and suppressing parasitic reactions, thus significantly improving the Coulombic efficiency of aqueous zinc metal batteries under low temperatures.
- At a low temperature of − 40 °C, the Zn||Zn cells maintained stable plating/stripping cycles for over 2500 h, and the Zn||PANI full cell exhibited excellent low-temperature performance with over 8000 charge–discharge cycles and a high capacity retention of more than 90%.

**Supplementary Information:**

The online version contains supplementary material available at 10.1007/s40820-025-01889-9.

## Graphical abstract

**Figure:**
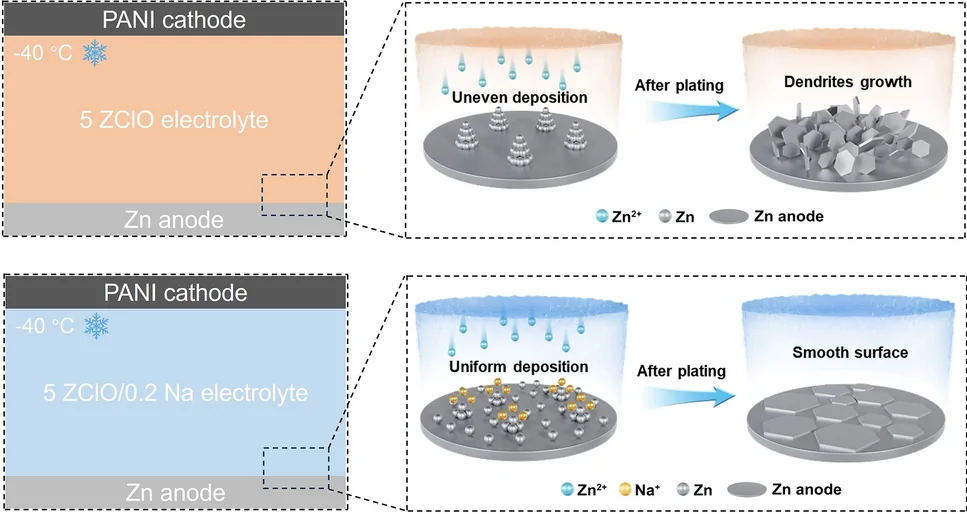

## Introduction

Aqueous zinc metal batteries (AZMBs) have emerged as highly promising next-generation energy storage systems for renewable energy applications, owing to their exceptional safety and high theoretical capacity [1]. The zinc metal anode offers abundant reserves and outstanding theoretical energy density (820 mAh g^−1^ and 5855 mAh cm^−3^), along with a favorable redox potential (− 0.76 V vs. SHE) [2]. Additionally, aqueous electrolytes exhibit high ionic conductivity, low volatility, and non-flammability [3]. These advantages make AZMBs become popular subject of research. However, AZMBs still face a series of challenges during low-temperature operation, including electrolyte freezing, uneven zinc deposition and dendrite growth due to slow ion transport, as well as hydrogen evolution reaction (HER) [4–8]. These issues can severely impair the low-temperature performance of aqueous batteries and restrict their application scenarios and commercialization process [9–12].

**Fig. 1:**
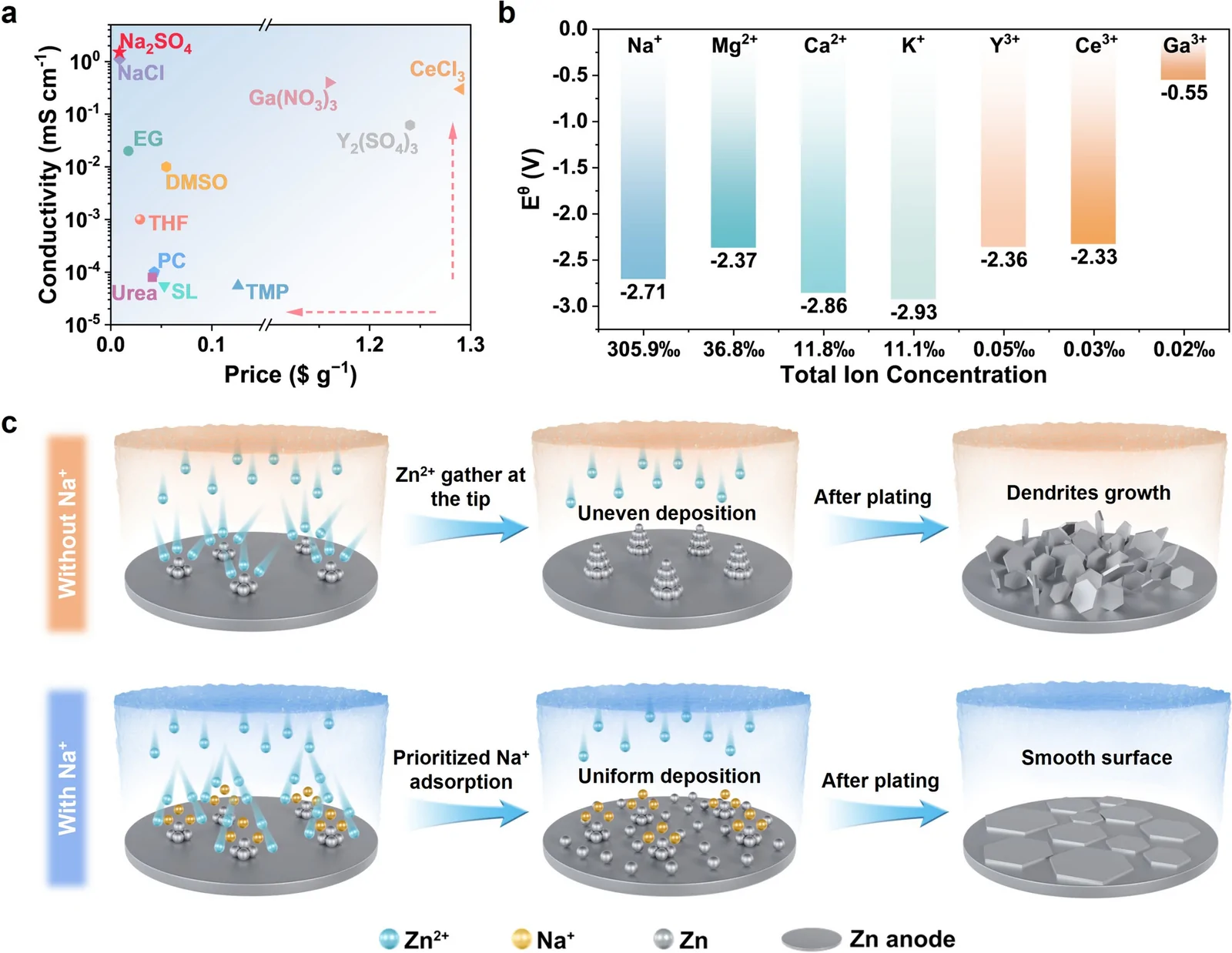
**a** Schematic illustration of the relationship between cost and ionic conductivity for reported electrolyte additives in aqueous solution at 25 °C. **b** Standard reduction potentials and natural abundances of representative metal cations, highlighting their suitability as electrolyte additives. **c** Proposed mechanism by which Na^+^ regulate Zn^2+^ distribution at the anode interface, suppress dendrite formation, and improve Zn deposition behavior under low-temperature conditions. (Color figure online)

Enhancing the low-temperature performance of AZMBs relies on improving the resistance of aqueous electrolytes to freezing. Organic additives like DMSO, EG, and SL can lower freezing points, but their weak polarity reduces ionic conductivity, increases viscosity, and impairs ion transport at low temperatures (Fig. 1a) [13–16]. To address these limitations, increasing the salt concentration has emerged as an alternative to avoid the drawbacks of organic additives. Organic-free electrolytes with strong antifreezing properties include 7.5 m ZnCl_2_ and 5 m Zn(ClO_4_)_2_, hereafter referred to as 5 ZClO [17–19]. The high-concentration ClO_4_^−^ in 5 ZClO has a strong electronegativity, which can effectively disrupt the water–water hydrogen bond network in the electrolyte [20–24]. This enables 5 ZClO to exhibit exceptional low-temperature resistance and high ionic conductivity as an aqueous electrolyte. While these electrolytes effectively enhance the freezing resistance of aqueous electrolytes, they overlook the critical challenges to anode stability induced by elevated salt concentrations. According to current theory, water molecules coordinated with cations in solvent-separated ion pairs (SSIP) are the primary source of HER. In dilute solutions, an increase in salt concentration leads to the formation of more SSIP, thereby enhancing HER activity. Additionally, higher cation concentrations may induce localized deposition, promoting dendrite growth, which is detrimental to the stability of the anode [25–29].

The incorporation of high-stability inorganic salts into organic-free antifreeze electrolytes enhances anode stability while maintaining high ionic conductivity and safety. Existing reports suggest that introducing metal cations that are less prone to deposition, such as Ce^3+^, Y^3+^, and Ga^3+^, can regulate the distribution of Zn^2+^ at the anode interface through electrostatic interactions, improving the uniformity of Zn^2+^ deposition and partially suppressing the HER [30–36]. Therefore, the introduction of such cations into conventional aqueous electrolytes represents an effective strategy to improve zinc anode stability. By comparing the content and reduction potential of reported metal cations, along with a comprehensive evaluation of the cost and safety of inorganic salt additives, Na_2_SO_4_ emerges as a promising additive owing to its affordability, relatively low reduction potential, and high ionic conductivity, making it a practical choice for enhancing the low-temperature performance of AZMBs (Fig. 1b).

Here, we develop a stable antifreezing aqueous electrolyte by introducing trace Na_2_SO_4_ into a highly concentrated 5 ZClO system. This modified electrolyte enables dendrite-free Zn deposition and suppressed hydrogen evolution reaction (HER), thereby achieving long-term low-temperature operation of aqueous Zn metal batteries. Spectroscopic characterizations, including Raman and infrared analysis, and molecular dynamics simulations, reveal that Na_2_SO_4_ further disrupts the water hydrogen bond network and enhances electrolyte structuring at subzero temperatures. In situ optical and atomic force microscopy further confirms that Na^+^ regulates interfacial Zn^2+^ distribution and inhibits dendrite formation under − 40 °C. We demonstrate that Na^+^, possessing a low reduction potential (− 2.71 V vs. SHE), can electrostatically repel Zn^2+^ at the anode interface, effectively mitigating tip-induced Zn^2+^ aggregation and uneven plating (Fig. 1c). This electrostatic interaction, revealed through density functional theory calculations, is key to suppressing Zn dendrites and HER at low temperatures. Benefiting from this design, Zn||Zn cells exhibit ultra-stable cycling over 2500 h at 5 mA cm^−2^ and 1 mAh cm^−2^ under − 40 °C. Even under high capacity conditions of 10 mAh cm^−2^ and 61% depth of discharge (DOD), Zn deposition remains stable for 360 h. Furthermore, Zn||Cu cells maintain a Coulombic efficiency above 99.5% for over 1000 cycles, and Zn||PANI full cells retain 91% of their capacity over 8000 cycles at − 40 °C. The design presented in this work provides a simple and cost-effective pathway toward high-performance, low-temperature AZMBs, offering new prospects for cold region energy storage applications.

## Experimental Section

### Materials Preparation of Sample

Zinc perchlorate hexahydrate (Zn(ClO_4_)_2_·6H_2_O, 99.9%), sodium sulfate anhydrous (Na_2_SO_4_, 99.5%), *N*-methyl-2-pyrrolidone (NMP, 99.7%), and polyaniline (PANI, 98%) were purchased from Sinopharm Chemical Reagent Co., Ltd. Zinc foil (thickness: 0.1 mm and 0.03 mm, purity: 99.99%) was used as the anode material. Other materials, including battery-grade poly(vinylidene fluoride) (PVDF), glass fiber separators, and Super P, were supplied by Guangdong Canrd New Energy Technology Co., Ltd. All chemicals were used as received without further purification.

### Material Characterizations

Composition, morphology, and structure of zinc anode were detected by X-ray diffraction (XRD Philips PC-APD), X-ray Photoelectron Spectroscopy (XPS VG ESCALAB 220i-XL), atomic force microscope (AFM Bruker Dimension ICON), and scanning electron microscopy (SEM SU8020). The morphology evolution of Zn anode during the plating process was detected by the in situ optical microscopy (OM LIB-XAS-SQ). The characteristics of electrolyte were conducted by differential scanning calorimeter (DSC-1), Raman spectroscopy (WITEC-CRM200 with 532 nm excitation laser, 400–4000 cm^−1^), attenuated total refraction–Fourier transform infrared spectroscopy (ATR-FTIR Spectrum Two, 400–4000 cm^−1^), and ^1^H nuclear magnetic resonance (^1^H NMR BRUKER AVANCE 400 MHz).

### Electrochemical Measurement

The polyaniline (PANI) electrode was prepared by mixing PANI, Super P, and PVDF binder in a weight ratio of 5:4:1. The mixture was ground for 40–50 min until homogeneous, followed by the addition of a few drops of NMP to form a uniform slurry. The slurry was coated onto carbon paper and dried at 60 °C for 6 h to obtain the electrode.

Electrochemical performance was evaluated using CR2032-type coin cells assembled with Zn||Zn cells, Zn||Cu cells, and Zn||PANI full cells. All tests were conducted on a Neware Battery Tester (CT-4000). For Zn||Zn and Zn||Cu cells, galvanostatic cycling was performed at a constant current density of 5 mA cm^−2^ with charge/discharge intervals of 1 h. The Zn||PANI full cells were tested within a voltage window of 0.2–1.4 V.

Electrochemical measurements including linear sweep voltammetry (LSV), cyclic voltammetry (CV), chronoamperometry (CA), Tafel analysis, and electrochemical impedance spectroscopy (EIS) were carried out using an electrochemical workstation (CHI760E, Shanghai ChenHua). The scan rate for CV and LSV was 5 mV s^−1^. Chronoamperometry was conducted at a constant potential of − 150 mV for 300 s in Zn||Zn cells. EIS measurements were performed using Zn||Zn cells equipped with two stainless steel spacers over a frequency range of 0.01 Hz to 10^5^ Hz. The electrolyte resistance (*Rs*, Ω) was determined from the high-frequency intercept of the Nyquist plot. Ionic conductivity (*σ*, in mS cm^−1^) was calculated using Eq. (1):

1 $$\sigma = G\frac{L}{A} = \frac{L}{{R_{s} A}}$$

where *σ* (mS cm^−1^) is the unit conductivity, *G* is the conductance, *Rs* (Ω) is the electrolyte resistance, *L* (cm) is the spacing distance between two stainless steel electrodes, and *A* (cm^−2^) is the area of the electrodes.

### Computational Methods

Molecular dynamics (MD) simulations: GROMACS software [37] was employed to perform the classical molecular dynamics simulations [38]. A cubic box of 50 Å was used, and periodic boundary conditions were set in all three directions. The OPC3 water model [39] and force fields from GAFF [40] for anions and Merz [41] for cations were applied. The atomic charges of anions were calculated using the Multiwfn software [42, 43] after structural optimization with ORCA 5.0.3 software package [44]. Electrostatic interactions were computed using the particle mesh Ewald (PME) method, with 12 Å cutoffs for both van der Waals and electrostatic interactions. The system was first equilibrated in the isothermal–isochoric (NVT) ensemble for 1 ns at 298 and 233 K, followed by isothermal isobaric (NPT) equilibration at 1 bar. A 10 ns production simulation in the NPT ensemble was performed for analysis. The temperature and pressure coupling were performed in V-rescale and C-rescale method, respectively.

Adsorption energy calculations: Density functional theory (DFT) calculations for adsorption energy were conducted with Vienna Ab Initio Simulation Package (VASP) [45]. Projector-augmented plane-wave (PAW) approach was used to describe the ion–electron interaction [46]. A generalized gradient approximation (GGA) expressed by the Perdew, Burke, and Ernzerhof functional was adopted [47]. And the self-consistent-field (SCF) calculations are finished until both the total energy difference between two iterations and the Hellman–Feynman forces on atoms are converged to within 1 × 10^−5^ eV and less than 0.02 eV Å^−1^, respectively. A 500 eV cutoff energy for the plane-wave basis set was found to correctly treat the valence electrons. A vacuum layer of 15 Å along the z direction was added to avoid interactions between periodically repeated images. Brillouin zone was sampled 3 × 3 × 1 gamma centered Monkhorst–Pack k-point grids. The VESTA software was used to generate Figs. 3a, b and S7, S8 [48].

Binding energy calculation: Binding energy calculations were performed using ORCA 5.0.3 software package [44]. The structure optimization and energy calculation among Zn^2+^, Na^+^, and H_2_O were performed using B3LYP/def2-TZVP [49]. The calculation formula of binding energy (*E*_b_) between water molecules and Zn^2+^ or Na^+^ is shown below:

2 $$E_{{\mathrm{b}}} = E_{{{\mathrm{complex}}}} - E_{{\mathrm{c}}} - E_{{\mathrm{m}}}$$

where *E*_complex_ is the total energy of the cation–water complex, *E*_c_ is the energy of the cation, *E*_m_ is the energy of the water molecule.

## Results and Discussion

### Antifreezing Performance and Characterization of Electrolytes

**Fig. 2:**
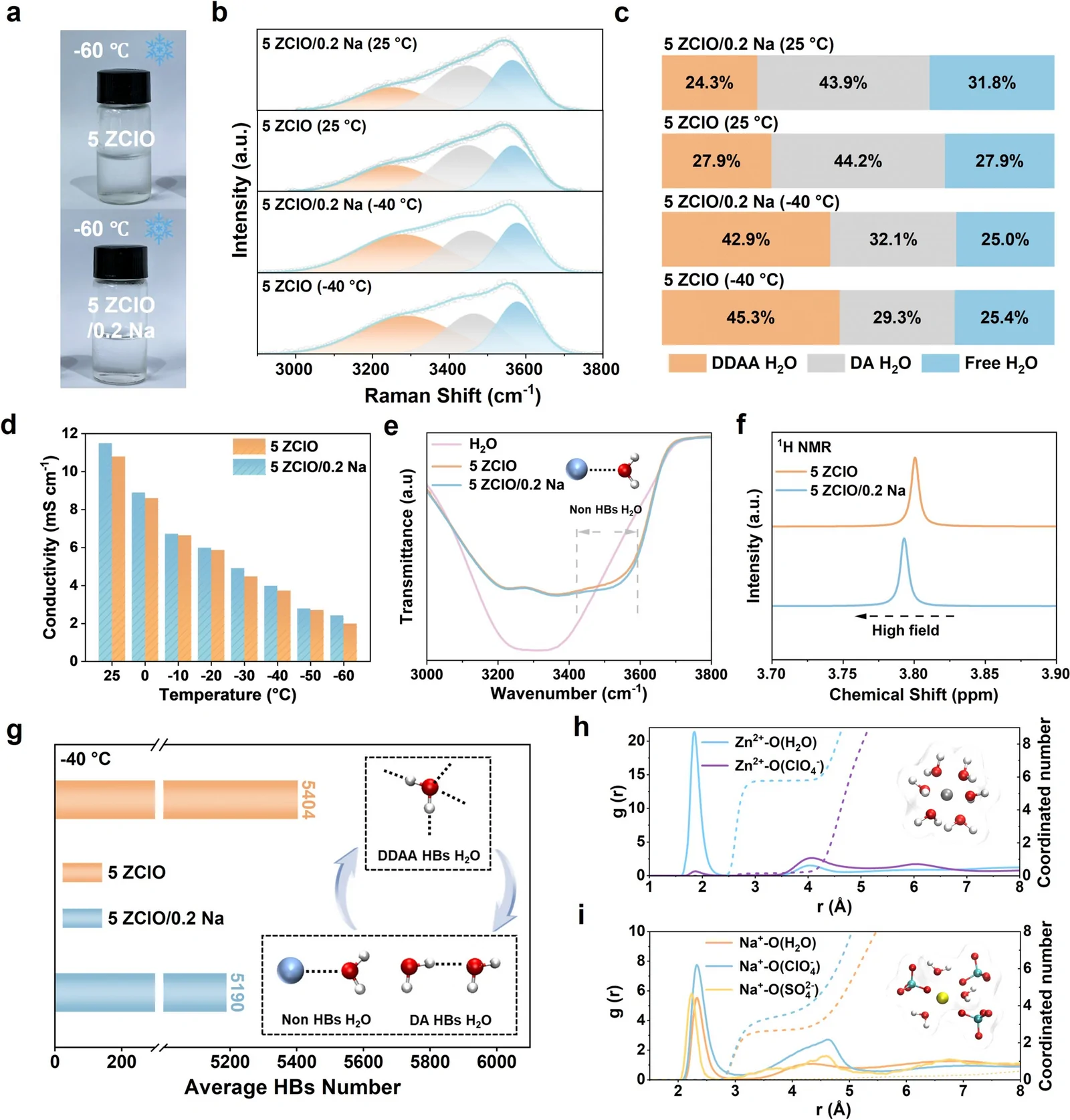
**a** Optical photographs of 5 ZClO and 5 ZClO/0.2 Na electrolytes stored under − 60 °C. **b** Raman signals of O–H stretching vibrations of two electrolytes at different temperatures. **c** Comparison of the areal ratios of different types of water electrolytes derived from fitting accumulated peaks between 2900 and 3800 cm^−1^ at different temperatures. **d** Ionic conductivities of the two electrolytes over the temperature range of 25 to − 60 °C. **e** FTIR spectra in the region from 3000 to 3800 cm^−1^ at room temperature. The involved color code in **e**: red O, white H, blue ion. **f** ^1^H NMR spectra of two electrolytes. **g** The average HBs number of water clusters in two electrolytes collected from MD simulations. **h** RDFs and schematic view of the primary solvation shell of Zn^2+^ in 5 ZClO and **i** solvation structure of Na^+^ in 5 ZClO/0.2Na. The involved color code in **h** and **i**: red O, white H, gray Zn, gold Na, cyan Cl. (Color figure online)

To avoid salt precipitation and keep the electrochemical stability of the electrolyte, 5 m Zn(ClO_4_)_2_ with 0.2 m Na_2_SO_4_ is selected as the optimized electrolyte formulation, denoted as 5 ZClO/0.2 Na. As shown in Figs. 2a and S3, there is no freezing or precipitation in both the 5 ZClO and 5 ZClO/0.2 Na electrolytes at − 60 °C. Therefore, we chose spectroscopic techniques to characterize the cryogenic stability of the electrolytes.

Water molecules in the solution can be categorized based on the number of hydrogen bond donors and acceptors: double donor–double acceptor (DDAA) type, single donor–single acceptor (DA) type, and non-hydrogen bonds (non-HBs) water molecules. The O–H bond Raman vibrational peaks for these three types of water molecules are observed at approximately 3250 cm^−1^ (DDAA-type water), 3450 cm^−1^ (DA-type water), and 3580 cm^−1^ (non-HBs type water), respectively [18]. Raman spectroscopy characterization results for both electrolytes show that whether at 25 or − 40 °C the Raman peak of 5 ZClO/0.2 Na electrolyte intensity at around 3250 cm^−1^ is lower, and the peak area proportion is smaller. Conversely, in the 5 ZClO/0.2 Na electrolyte, the Raman peak intensity at around 3580 cm^−1^ is higher, and the peak area proportion is larger (Fig. 2b, c). This indicates that the addition of Na_2_SO_4_ promotes the conversion of some DDAA-type water molecules into non-HBs type water. DDAA-type water molecules serve as building blocks for ice crystal formation, whereas non-HBs water molecules represents dynamic species associated with ion hydration [50]. Compared to the 5 ZClO electrolyte, the 5 ZClO/0.2 Na system exhibited a lower proportion of DDAA-type water and a higher proportion of non-HBs type water at both 25 and − 40 °C, indicating its enhanced freeze resistance. At the same time, we tested the ion conductivity of the electrolyte at different temperatures, which is a critical parameter. The results shown in Fig. 2d demonstrate that in the wide temperature range from 25 to − 60 °C, the ion conductivity of 5 ZClO/0.2 Na is higher than that of 5 ZClO. This improvement is due to the further disruption of the electrolyte’s hydrogen bonds network, allowing cations to be transmitted more quickly.

The ATR-FTIR characterization results of the electrolyte indicate that the absorption peak area of non-HBs type water at 3580 cm^−1^ significantly increases after adding Na_2_SO_4_, suggesting an increased proportion of water molecules which have not formed hydrogen bonds in the electrolyte (Fig. 2e), corroborating the Raman data. The ^1^H NMR can also be used to study hydrogen bonding in the electrolyte. In the ^1^H NMR of the two electrolytes, it is observed that the ^1^H peak chemical shift decreases after adding Na_2_SO_4_, indicating a weakened shielding effect of the hydrogen atom’s electron cloud, which means that the overall hydrogen bonding in the electrolyte is reduced and weakened (Fig. 2f). Moreover, the Raman peak of the Cl–O bond at 933 cm^−1^ shows no shift at either 25 or − 40 °C, suggesting that temperature variations do not affect the coordination between ClO_4_^−^ and Zn^2+^ (Fig. S4). This implies that temperature has no significant impact on the solvation structure of Zn^2+^.

MD simulations of the average number of hydrogen bonds in the electrolyte shows that compared to 5 ZClO, 5 ZClO/0.2 Na exhibits a lower overall average number of hydrogen bonds. As shown in Fig. 2g, the reduction in the average number of hydrogen bonds is attributed to the addition of Na_2_SO_4_, which causes more DDAA-type water molecules to transform into DA-type water molecules or form non-HBs water molecules coordinated with cations. The average number of hydrogen bonds can correspond to the Raman test results mentioned above. To better investigate the role of electrolyte additives at the microscopic level, we performed radial distribution functions (RDF) and coordination number analyses. The results show that at both 25 and − 40 °C, the solvation shell of Zn^2+^ in 5 ZClO contains approximately six water molecules. In the case of 5 ZClO/0.2 Na, the solvation shell of Zn^2+^ is also composed of approximately six water molecules (Figs. 2h and S9). However, at both 25 and − 40 °C the addition of Na^+^ leads to the adsorption of three additional water molecules, forming its own solvation shell (Figs. 2i and S9). This indicates that the introduction of Na⁺ increases the proportion of non-HBs water molecules in the electrolyte, confirming the previous explanation.

### Preferential Adsorption Mechanism of Na^+^ and Its Role in Improving Zn^2+^ Deposition

**Fig. 3:**
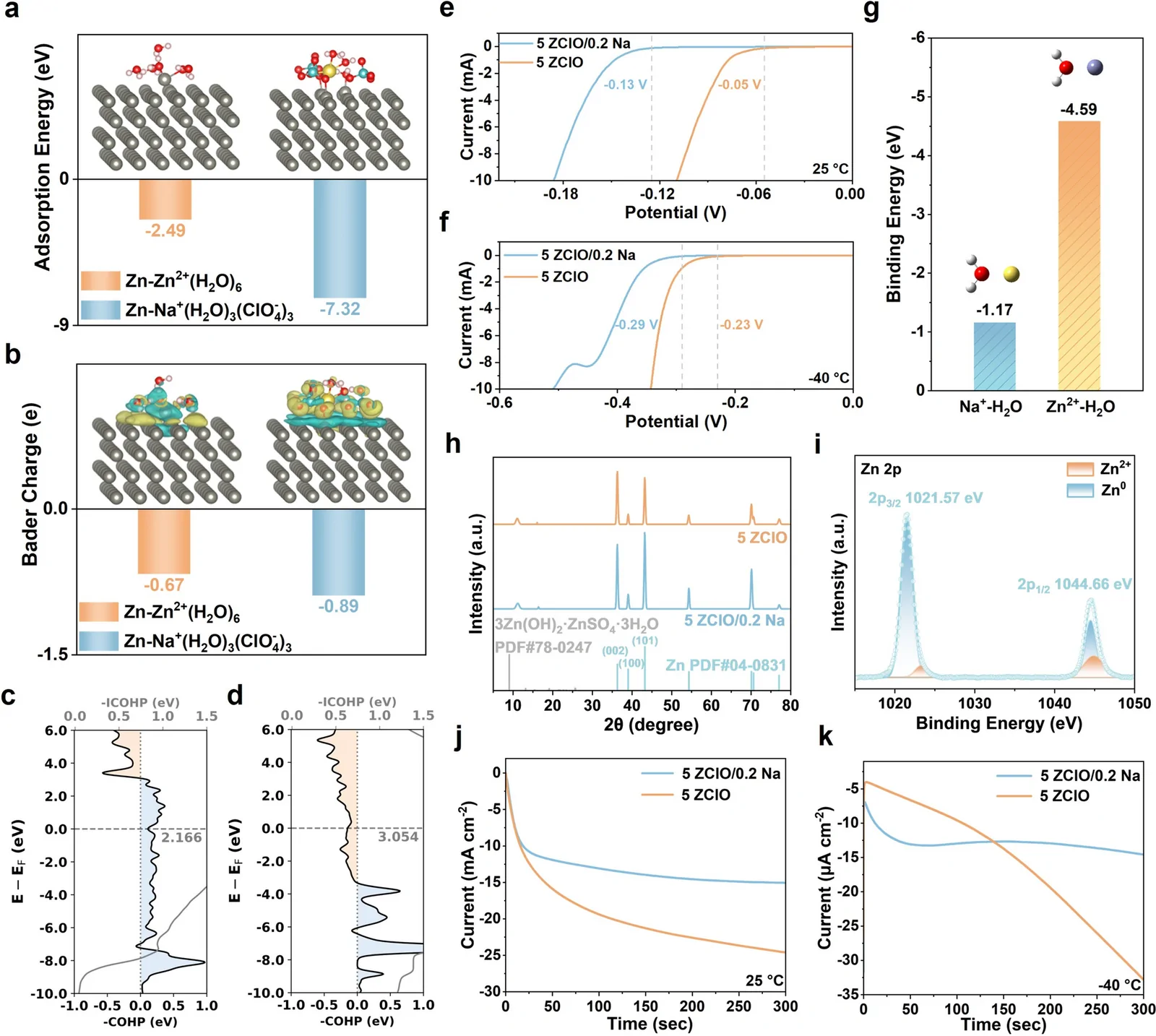
**a** Adsorption energy of the hydrated cations on zinc surface. **b** Charge density difference for demonstrating the interfacial adsorption model of the cathode for solvation structures. The involved color code: red O, white H, gray Zn, gold, Na cyan Cl. **c**, **d** Crystal orbital Hamilton population of the hydrated cations on zinc surface. **e**, **f** HER investigation in the different electrolytes at 25 °C and − 40 °C. **g** Binding energies of different cations with water molecules. **h** XRD patterns of Zn anodes after cycling 100 cycles at a current density of 5 mA cm^−2^. **i** XPS spectra of Zn anodes after cycling 100 cycles at a current density of 5 mA cm^−2^. **j** CA curves of Zn anodes in different electrolytes at 25 °C. **k** CA curves of Zn anodes in different electrolytes at − 40 °C. (Color figure online)

In aqueous electrolytes, metal cations typically exist as hydrated ions. Thus, to investigate the preferential adsorption behavior of sodium ions on the zinc anode, a comparative analysis of the adsorption energies of solvated Na^+^ and Zn^2+^ ions on the zinc metal anode was conducted. According to RDF results, Zn^2+^ is predominantly present as Zn^2+^(H_2_O)_6_ across different temperatures in both electrolytes, while Na⁺ is present as Na^+^(H_2_O)_3_(ClO_4_^−^)_3_ (Fig. 2h, i). DFT calculations show that the adsorption energy of Na^+^(H_2_O)_3_(ClO_4_^−^)_3_ on the Zn metal anode is as high as − 7.32 eV, significantly greater than the adsorption energy of Zn^2+^(H_2_O)_6_ on the Zn anode which is − 2.49 eV (Fig. 3a). This indicates that Na^+^(H_2_O)_3_(ClO_4_^−^)_3_ preferentially adsorbs onto the surface of the Zn anode. Additionally, due to the higher reduction potential of Na^+^, it does not deposit but instead continuously provides electrostatic repulsion to Zn^2+^ on the anode surface, preventing the aggregation of Zn^2+^(H_2_O)_6_ and promoting uniform deposition. Moreover, the differential charge density of Na^+^(H_2_O)_3_(ClO_4_^−^)_3_ on the zinc metal anode surface is higher than that of Zn^2+^(H_2_O)_6_, and the Bader charge transfer is also greater (Fig. 3b). In the crystal orbital Hamilton population (COHP) analysis of Na^+^(H_2_O)_3_(ClO_4_^−^)_3_, the proportion of bonding orbitals is larger, and the integrated bonding orbital value is higher as well (Fig. 3c, d). These results indicate that, compared to Zn^2+^(H_2_O)_6_, Na^+^(H_2_O)_3_(ClO_4_^−^)_3_ has a stronger tendency to adsorb onto the surface of the zinc metal anode.

After confirming that Na^+^ preferentially adsorbs onto the anode, we further investigate how Na⁺ enhances anode stability. As shown in Figs. 3d, e and S9, S10, both LSV and tafel test results indicate that, across a temperature range of 25 to − 60 °C, Zn||Zn cells assembled with the 5 ZClO/0.2 Na electrolyte exhibit a higher onset potential for HER and a lower corrosion current. This implies that the 5 ZClO/0.2 Na electrolyte effectively suppresses HER, thereby reducing side reactions on the anode. RDF and adsorption energy results have shown that fewer water molecules are coordinated with Na^+^ than with Zn^2+^. Additionally, once Na^+^ adsorbs preferentially onto the anode, it reduces the aggregation of hydrated Zn^2+^ on the anode through electrostatic repulsion, consequently decreasing the number of coordinated water molecules at the anode interface. As mentioned in introduction, the water molecules coordinated with cations are the primary source of HER. The introduction of Na^+^ reduces the formation of coordinated water molecules at the anode interface, thereby suppressing the HER activity of the electrolyte.

Furthermore, by calculating the binding energies between Na^+^, Zn^2+^, and water molecules, we find that Na^+^ has a binding energy with water of only − 1.17 eV, whereas Zn^2+^ has a binding energy of − 4.59 eV with water (Fig. 3g). This is because the Zn^2+^ has a smaller radius than the Na^+^ and carries a greater charge, resulting in a stronger attraction to the polar water molecules, which suggests that Na^+^ exerts a weaker pull on water molecule, meaning the O–H bonds in water molecules coordinated with Na^+^ are less disturbed, more stable, and less prone to decomposition [51]. These combined factors contribute to the reduced HER activity in the 5 ZClO/0.2 Na electrolyte. Additionally, we extracted the anodes from Zn||Zn cells assembled with 5 ZClO and 5 ZClO/0.2 Na electrolytes after 100 charge–discharge cycles to perform XRD and XPS analyses. These tests aimed to identify any byproducts formed on the anode post-cycling. XRD results confirmed that, after introducing Na_2_SO_4_ into 5 ZClO and cycling for 100 times, no harmful byproducts such as Zn_4_SO_4_(OH)_6_·5H_2_O were observed on the anode surface (Figs. 3h and S12). XPS results also indicated a very low S content on the cycled anode, further suggesting that almost no Zn_4_SO_4_(OH)_6_·5H_2_O formed (Figs. 3i and S13). This demonstrates that SO_4_^2−^ did not negatively impact the deposition process.

To verify the role of Na^+^ in regulating uniform Zn^2+^ deposition, we conducted CA tests on Zn||Zn cells with different electrolytes. A constant voltage of − 150 mV was applied, and the current response was recorded over a 300-s interval. As shown in Fig. 3j, k, at both 25 and − 40 °C, the cell using the 5 ZClO/0.2 Na electrolyte exhibited a quicker stabilization of current and maintained a stable current throughout the remaining period. In contrast, the cell using the 5 ZClO electrolyte showed a continuous increase in current, struggling to achieve stability. This indicates that Zn^2+^ in the 5 ZClO/0.2 Na electrolyte rapidly entered a uniform deposition phase under constant voltage, maintaining a steady deposition rate. These observations confirm that the presence of Na^+^ aids in modulating deposition current flux, thereby preventing excessive localized Zn^2+^ deposition. The data obtained from the CA test were normalized to create a (*I*/*I*_m_)^2^ − (*t*/*t*_m_) dimensionless curve and analyzed using the Scharifker–Hills model [52], which describes two nucleation modes:

3 $$\begin{array}{*{20}c} {\left( {\frac{I}{{I_{{\mathrm{m}}} }}} \right)^{2} = \frac{1.9542}{{\left( {\frac{t}{{t_{{\mathrm{m}}} }}} \right)}} \left\{ {1 - \exp \left[ { - 1.2564\left( {\frac{t}{{t_{{\mathrm{m}}} }}} \right)} \right]} \right\}^{2} \quad ({\mathrm{instantaneous}}\,{\mathrm{nucleation}}) } \\ \end{array}$$

4 $$\begin{array}{*{20}c} {\left( {\frac{I}{{I_{{\mathrm{m}}} }}} \right)^{2} = \frac{1.2254}{{\left( {\frac{t}{{t_{{\mathrm{m}}} }}} \right)}} \left\{ {1 - \exp \left[ { - 2.3367\left( {\frac{t}{{t_{{\mathrm{m}}} }}} \right)^{2} } \right]} \right\}^{2} \quad ({\mathrm{progressive}}\,{\mathrm{nucleation}})} \\ \end{array}$$

As shown in Fig. S14, that at 25 °C, the dimensionless curve for the deposition with 5 ZClO/0.2 Na electrolyte aligns more closely with the instantaneous nucleation curve. In contrast, the dimensionless curve for the deposition with 5 ZClO falls between the instantaneous nucleation and progressive nucleation curves, indicating the presence of both nucleation orientations, which is not conducive to forming more uniform zinc nuclei. At − 40 °C, the dimensionless curves for both electrolytes are significantly higher than the instantaneous nucleation curve, indicating that at low temperatures, both electrolytes follow a higher nucleation rate associated with instantaneous nucleation (Fig. S14). In general, instantaneous nucleation corresponds to fast nucleation that exhausts all nucleation sites at the very beginning of plating, while progressive nucleation is relatively sluggish and takes a longer time to occupy the nucleation sites. According to classical nucleation theory, under the same nucleation conditions, the number of nucleation sites is proportional to the cube of nucleation overpotential (*N* ∝ *η*^3^) [53]. This means that the larger the nucleation overpotential, the more nucleation sites will form, favoring denser and more uniform nucleation [54]. By assembling Zn||Cu cells with the two different electrolytes and conducting deposition/stripping cycle tests and CV tests, it was observed that, both at room temperature and low temperature, the cells with added Na_2_SO_4_ exhibit a larger nucleation overpotential during charging (Figs. S15 and S16). This means that the 5 ZClO/0.2 Na electrolyte forms more uniform and numerous zinc nuclei during nucleation. This occurs because Na^+^ inhibits the excessive deposition of Zn^2+^, requiring a higher initial potential for Zn^2+^ deposition, thus increasing the nucleation overpotential and promoting denser nucleation of zinc metal.

### Comparison of Anode Stability and Reversibility in Different Electrolytes

**Fig. 4:**
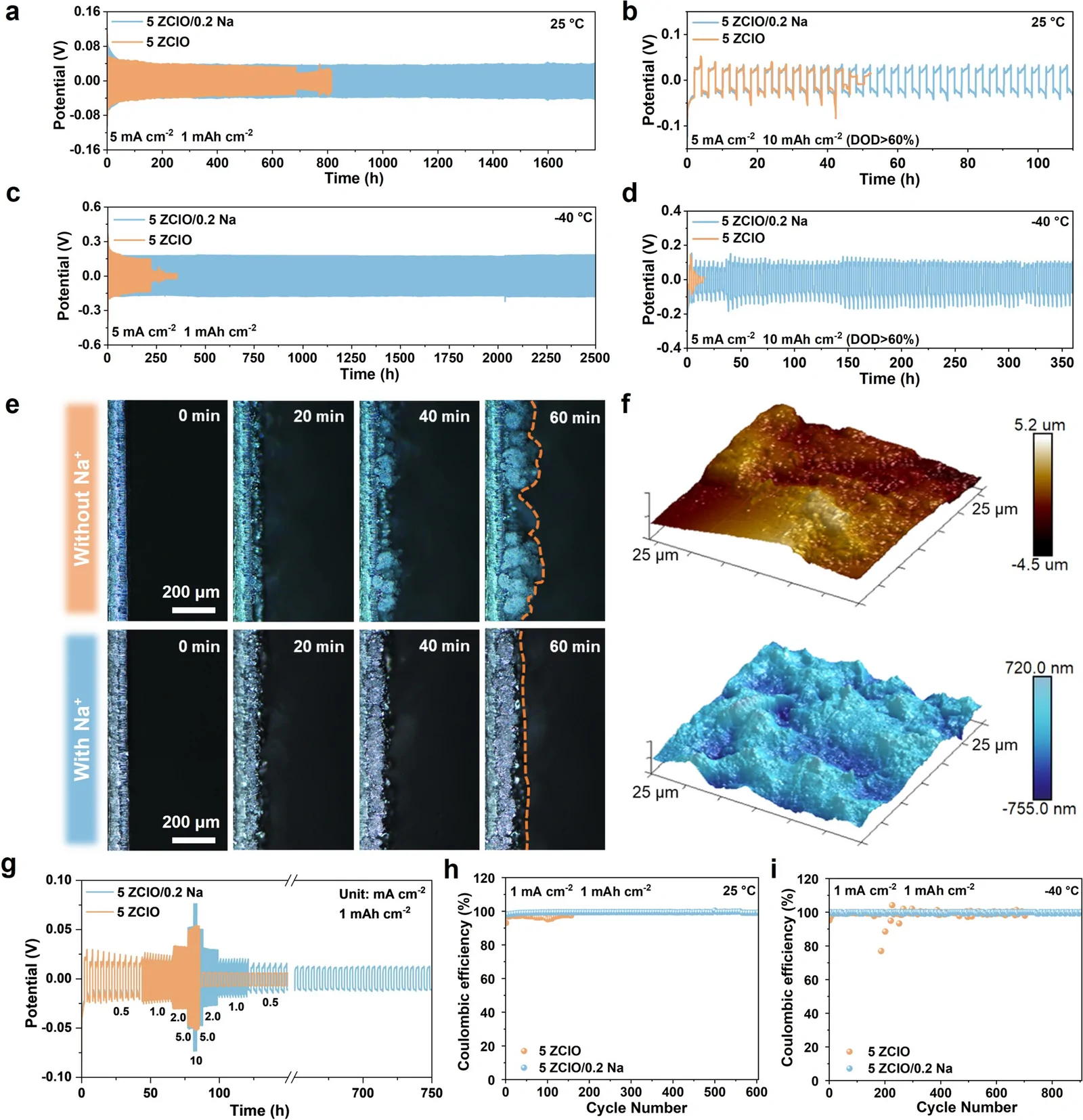
Cycling performance of Zn||Zn cells with or without Na_2_SO_4_ addition collected at **a** 25 °C, 1 mAh cm^−2^ and **b** 25 °C, 10 mAh cm^−2^ and **c** − 40 °C, 1 mAh cm^−2^ and** d** − 40 °C, 10 mAh cm^−2^. **e** In situ observations of zinc deposition in different electrolytes. **f** AFM images of the cycled Zn in different electrolytes. **g** Rate performances for the Zn||Zn cells with different electrolytes. **h** Zn plating/stripping CE at 25 °C in different electrolytes. **i** Zn plating/stripping CE at − 40 °C in different electrolytes. (Color figure online)

Due to the excellent low-temperature tolerance and anode stability of the 5 ZClO/0.2 Na electrolyte, cells using this electrolyte demonstrate highly reversible performance at both room and low temperatures. Zn||Zn cells assembled with 5 ZClO/0.2 Na electrolyte and 0.1-mm-thick zinc foil, cycled at a current density of 5 mA cm^−2^ and areal capacity density of 1 mAh cm^−2^, achieve a cycle life close to 1800 h at 25 °C and 2500 h at − 40 °C (Fig. 4a, b). When maintaining the current density but increasing the areal capacity density to 10 mAh cm^−2^, Zn||Zn cells assembled with 5 ZClO/0.2 Na electrolyte and 0.03-mm-thick zinc foil achieve a cycle life of 110 h at 25 °C and over 360 h at − 40 °C at a DOD exceeding 60%, significantly outperforming cells using 5 ZClO electrolyte (Fig. 4c, d). Rate performance tests were conducted on Zn||Zn cells with different electrolytes under varying current densities, revealing that the 5 ZClO/0.2 Na electrolyte exhibited superior current rate stability (Fig. 4g).

SEM images of the zinc metal anode after 100 charge/discharge cycles with different electrolytes reveal distinct morphological differences: Noticeable zinc dendrites are observed on the anode surface cycled with 5 ZClO, whereas the anode surface cycled with 5 ZClO/0.2 Na appears comparatively smooth and flat (Fig. S17). To further visually assess the effectiveness of 5 ZClO/0.2 Na electrolyte in suppressing dendrite growth, we conducted OM observations of the anode morphology under different electrolytes, setting a charging current density of 10 mA cm^−2^ and maintaining it for 1 h. The OM results indicate that the zinc anode in 5 ZClO quickly developed uneven zinc deposition, forming small protrusions that continued to grow throughout the charging process, eventually forming noticeable dendrites. In contrast, the zinc anode in 5 ZClO/0.2 Na electrolyte maintained uniform zinc deposition, exhibiting a smooth and flat surface throughout the 1-h charging process (Fig. 4e). After SEM observations, the zinc anode surfaces were further analyzed with atomic force microscopy to assess surface roughness. It was found that the zinc anode cycled in 5 ZClO displayed significant roughness, with a vertical height difference of up to 9.7 µm, while the zinc anode cycled in 5 ZClO/0.2 Na showed only a 1.5 µm height difference (Fig. 4f). These microscopic images confirm that the 5 ZClO/0.2 Na electrolyte effectively inhibits dendrite growth, thereby extending battery life.

To further demonstrate the effects of the 5 ZClO/0.2 Na electrolyte on the reversibility of Zn plating and stripping, Zn||Cu cells were assembled and subjected to testing. In half-cell cycling tests at a current density of 1 mA cm^−2^ and an areal capacity of 1 mAh cm^−2^ at 25 °C, the initial Coulombic efficiency (CE) of the 5 ZClO-based cell was only 91%, and it short-circuited after 180 cycles. In contrast, the 5 ZClO/0.2 Na-based half-cell achieved an initial CE of 94% at 25 °C, maintaining over 99.5% CE for 600 cycles (Fig. 4h). At − 40 °C, the 5 ZClO-based half-cell exhibited substantial CE fluctuations and instability, while the 5 ZClO/0.2 Na-based half-cell achieves stability and high stripping/plating efficiency with a remarkable average CE of 99.7% after 900 cycles (Fig. 4i). These Zn||Cu cell performance comparisons demonstrate that the 5 ZClO/0.2 Na electrolyte significantly suppresses side reactions, enhancing the reversibility and lifespan of the cell during low-temperature operation.

### Electrochemical Performance of Zn||PANI Full Batteries

Given that 5 ZClO/0.2 Na is primarily designed to meet the demands of extreme low-temperature environments, assembling full cells with 5 ZClO/0.2 Na electrolyte and testing them at low temperatures are central to this research. The performance of full cells at low temperatures is closely related to the choice of cathode material; traditional metal oxide cathodes and Prussian blue analogs typically face challenges with ion intercalation and deintercalation at low temperatures [55]. Organic cathodes such as PANI can store/release Zn^2+^ through dynamic coupling/decoupling processes, achieving the effect of storing and releasing electrical energy. This energy storage mechanism, which does not require an intercalation process, allows the PANI cathode to maintain fast ion kinetics even at extreme low temperatures [17]. Therefore, PANI was selected as the cathode material for assembling both full cells and pouch cells.

**Fig. 5:**
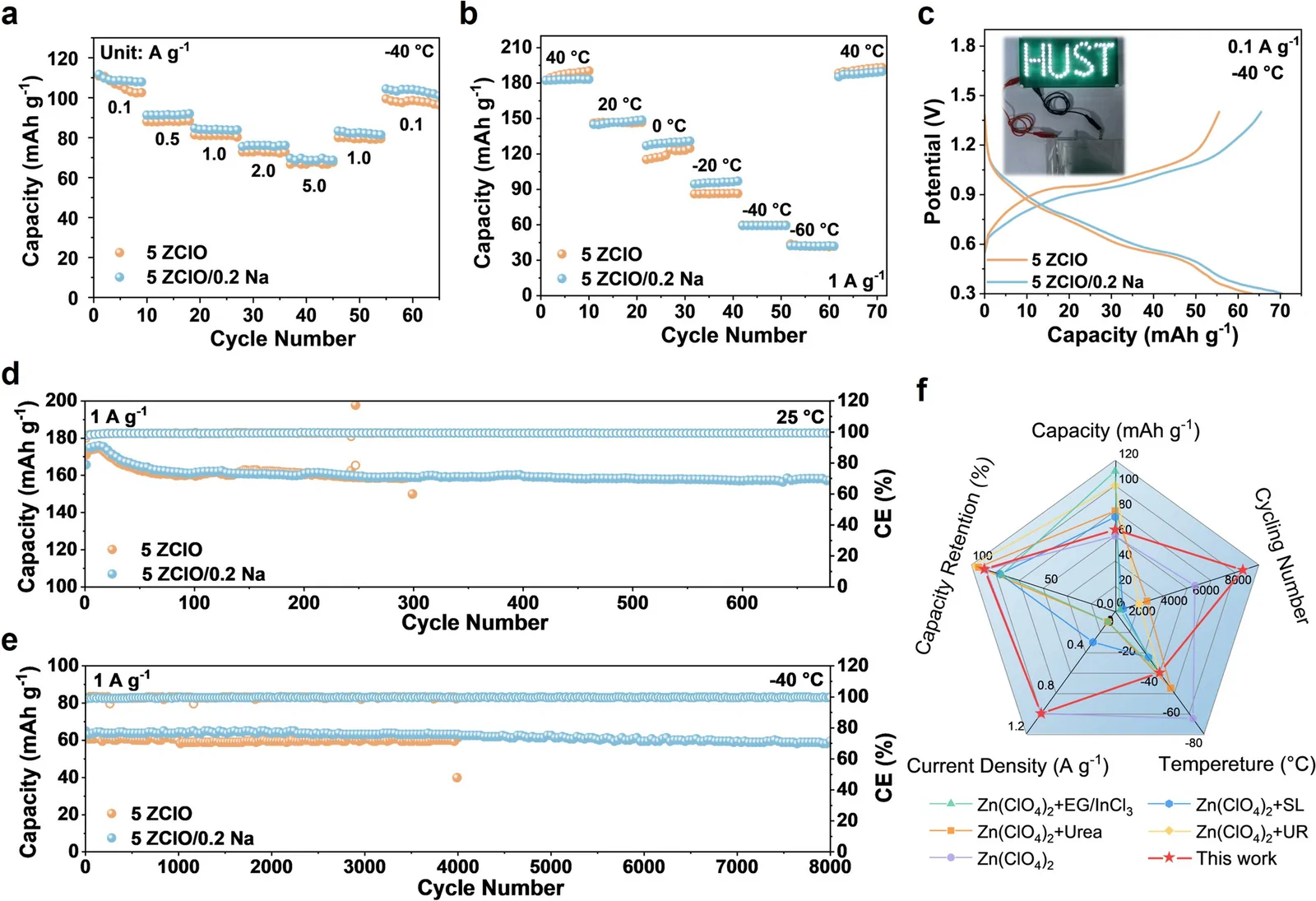
**a** Rate performance of Zn||PANI full batteries at different current densities at − 40 °C. **b** The capacity of Zn||PANI full batteries in the temperature range of 40 to − 60 °C. **c** Charge–discharge curves of pouch cells with different electrolytes. **d** Cycling performance of Zn||PANI full batteries at 25 °C. **e** Cycling performance of Zn||PANI full batteries at − 40 °C. **f** Low-temperature performance comparison of this work with previously reported ZIB systems. (Color figure online)

Charge–discharge tests were conducted on Zn||PANI full cells using different electrolytes at various temperatures and current densities to assess the electrolyte’s stability and low-temperature performance. Results indicate that full cells with the 5 ZClO/0.2 Na electrolyte exhibit superior capacity retention and temperature adaptability across a broad temperature range from 40 to − 60 °C (Fig. 5b). Even at − 40 °C, these cells demonstrate excellent rate performance; after increasing the current density from 0.1 to 5 mA cm^−2^ and then returning to 0.1 mA cm^−2^, the specific capacity nearly returns to its initial level (Fig. 5a). The full batteries assembled with the 5 ZClO/0.2 Na electrolyte are capable of stable cycling for over 700 cycles at 25 °C (Fig. 5d). At a low temperature of − 40 °C, the full battery still exhibits a high capacity of 65 mAh g^−1^ at a current density of 1 A g^−1^, maintaining stable operation for more than 8000 cycles. Even after 8000 cycles, the capacity retention remains greater than 90% (Fig. 5e). This performance is significantly superior to that of the full battery assembled with 5 ZClO, both in terms of cycle life and capacity retention. Compared with the pouch cell using 5 ZClO electrolyte, the pouch cell using 5 ZClO/0.2 Na electrolyte also exhibits higher specific capacity and longer operation life at low temperatures. (Figs. 5c and S20). The performance achieved in this study in terms of working temperature, current density, capacity, and cycling stability is compared with existing AZMBs and other batteries in Fig. 5f [56–58]. Our fabricated AZMBs delivered the best low-temperature performance with respect to capacity retention and cycling stability, exhibiting enormous potential in extreme environments.

## Conclusions

In summary, this work introduces a trace amount of low-cost Na_2_SO_4_ as an inorganic salt additive into the 5 ZClO electrolyte to form an organic-free 5 ZClO/0.2Na electrolyte, aimed at improving the cycling stability of AZMBs at low temperatures. Spectroscopic characterizations and MD simulation results demonstrate that adding Na_2_SO_4_ to the 5 ZClO further disrupts the water-water hydrogen bond network, thereby enhancing the antifreeze capability of the electrolyte and facilitating rapid Zn^2+^ transport at low temperatures. Microscopic characterizations and DFT calculations reveal that the introduced Na^+^ spontaneously adsorb onto the Zn anode surface, providing electrostatic repulsion for the subsequently deposited hydrated Zn^2+^, thereby inducing the uniform deposition of Zn^2+^. Additionally, Na^+^ reduces the number of water molecules coordinated with cations, thereby suppressing the HER and inhibiting the formation of harmful byproducts on the zinc metal anode. This work achieved stable plating/stripping cycles for Zn||Zn cells at − 40 °C for over 2500 h under conditions of 5 mA cm^−2^ and 1 mAh cm^−2^. Additionally, under conditions of 5 mA cm^−2^ and 10 mAh cm^−2^ with a over 60% DOD, stable plating/stripping cycles were maintained for 360 h. The Cu|| Zn cells maintained a coulombic efficiency of 99.5% for more than 1000 cycles at 1 mA cm^−2^, while Zn||PANI full cells achieved stable charge/discharge cycles for over 8000 cycles at a current density of 1 A g^−1^. This work providing a new strategy for enhancing the long-term operational stability of AZMBs at low temperatures.

## Supplementary Information

Below is the link to the electronic supplementary material.

Supplementary file1 (DOCX 7029 KB)
