# Supplementary material for: Prioritized Na+ Adsorption-Driven Cationic Electrostatic Repulsion Enables Highly Reversible Zinc Anodes at Low Temperatures
Source: Nanomicro Lett. 2025 Sep 1;18:47. doi: 10.1007/s40820-025-01889-9 (PMC12401825; doi:10.1007/s40820-025-01889-9)
Supplement: Supplementary file 1 — Supplementary file1 (DOCX 7029 KB) [file 40820_2025_1889_MOESM1_ESM.docx]

Supporting Information for

**Prioritized Na^+^ Adsorption-Driven Cationic Electrostatic Repulsion Enables Highly Reversible Zinc Anodes at Low Temperatures**

Guanchong Mao^1#^, Pan Xu^1#^, Xin Liu^1*^, Xingyu Zhao^1^ and Zexiang Shen^1^, Dongliang Chao^2^ Minghua Chen^1*^

^1^ Key Laboratory of Engineering Dielectric and Applications (Ministry of Education), School of Electrical and Electronic Engineering, Harbin University of Science and Technology, Harbin 150080, P. R. China

^2^ Laboratory of Advanced Materials, Aqueous Battery Center, Shanghai Key Laboratory of Molecular Catalysis and Innovative Materials, Electron Microscope Center of Fudan University, Shanghai Wusong Laboratory of Materials Science, and Faculty of Chemistry and Materials, Fudan University, Shanghai 200433, P. R. China

#Guanchong Mao and Pan Xu have contributed equally to this work.

*Corresponding authors. E-mail: [liu.xin@hrbust.edu.cn](mailto:liu.xin@hrbust.edu.cn) (Xin Liu); [mhchen@hrbust.edu.cn](mailto:mhchen@hrbust.edu.cn) (Minghua Chen)

**Supplementary Note**

Since the advantage of aqueous zinc metal batteries lies in their high safety, this work first excluded flammable and explosive chemicals when screening additives. NaClO_4_ as a dangerous chemical listed in the explosive precursor directory, was still excluded from our selection. Although it is a sodium salt that does not introduce other anions.

In this work, the zinc foil used for the charge-discharge cycle test with a high depth of discharge has a thickness of 0.03 mm. It was cut into regular circular electrode sheets with a diameter of 12 mm. Each zinc metal electrode sheet was weighed to be 0.0225 g, theoretically capable of providing a capacity of 18.45 mAh. The electrode sheet area is 1.13 cm^2^. The test conditions for the high-depth-of-discharge charge-discharge cycle test are an areal capacity of 10 mAh cm^−2^, meaning both the charge and discharge capacities are 11.3 mAh, accounting for approximately 61% of the theoretical capacity. Therefore, we consider that the discharge depth in the test conducted under the above conditions is greater than 60%.

**Supplementary Figures**

**
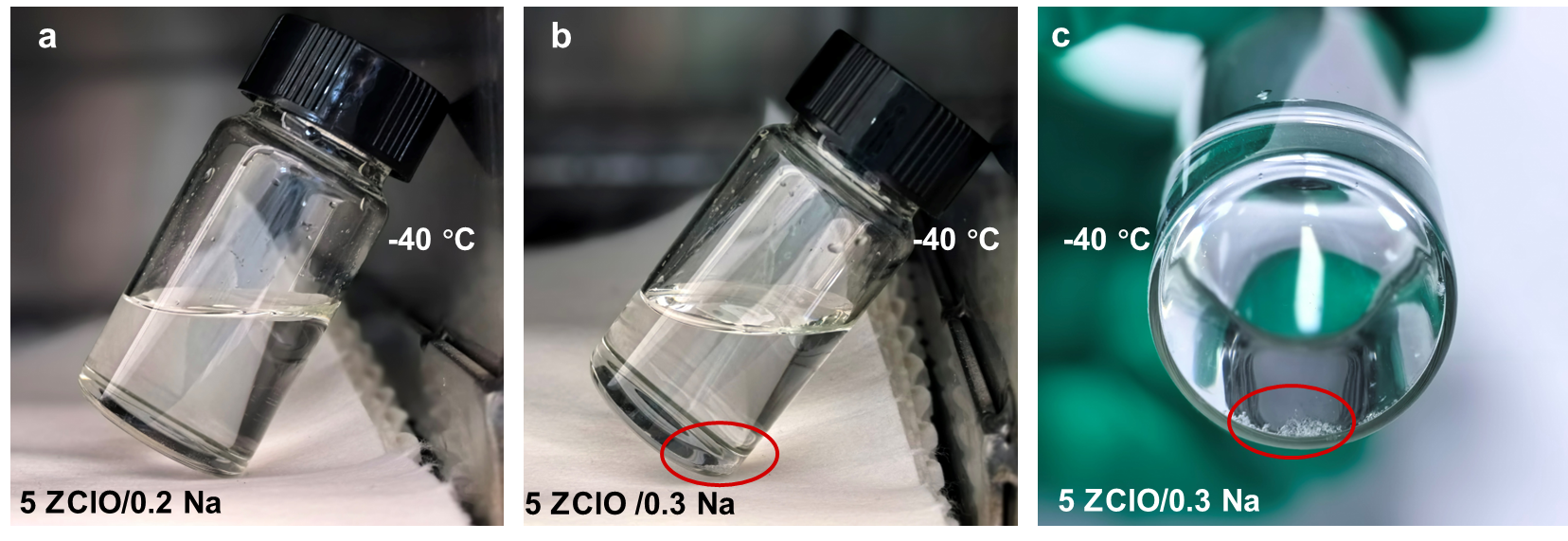
**

**Fig. S1** Optical photos of (**a**) 5 ZClO/0.2 Na, (**b**) and (**c**) 5 ZClO/0.3 Na electrolytes stored under −40 °C

Given the relatively low solubility of Na_2_SO_4_ at low temperatures, we established a 0.1 m concentration gradient to systematically determine the optimal additive concentration of Na_2_SO_4_. When 0.3 m Na_2_SO_4_ was added, salt precipitation occurred in the electrolyte at −40°C, while the addition of 0.1 m Na_2_SO_4_ exhibited no improvement in electrochemical performance (Figs. S1 and S2). Notably, only the 0.2 m Na_2_SO_4_ additive achieved a balance by avoiding salt precipitation and demonstrating superior electrochemical stabilit. Consequently, the optimal Na_2_SO_4_ concentration was identified as 0.2 m, and the optimized electrolyte formulation was determined to be 5 m Zn(ClO_4_)_2_ with 0.2 m Na_2_SO_4_, designated as 5 ZClO/0.2 Na.


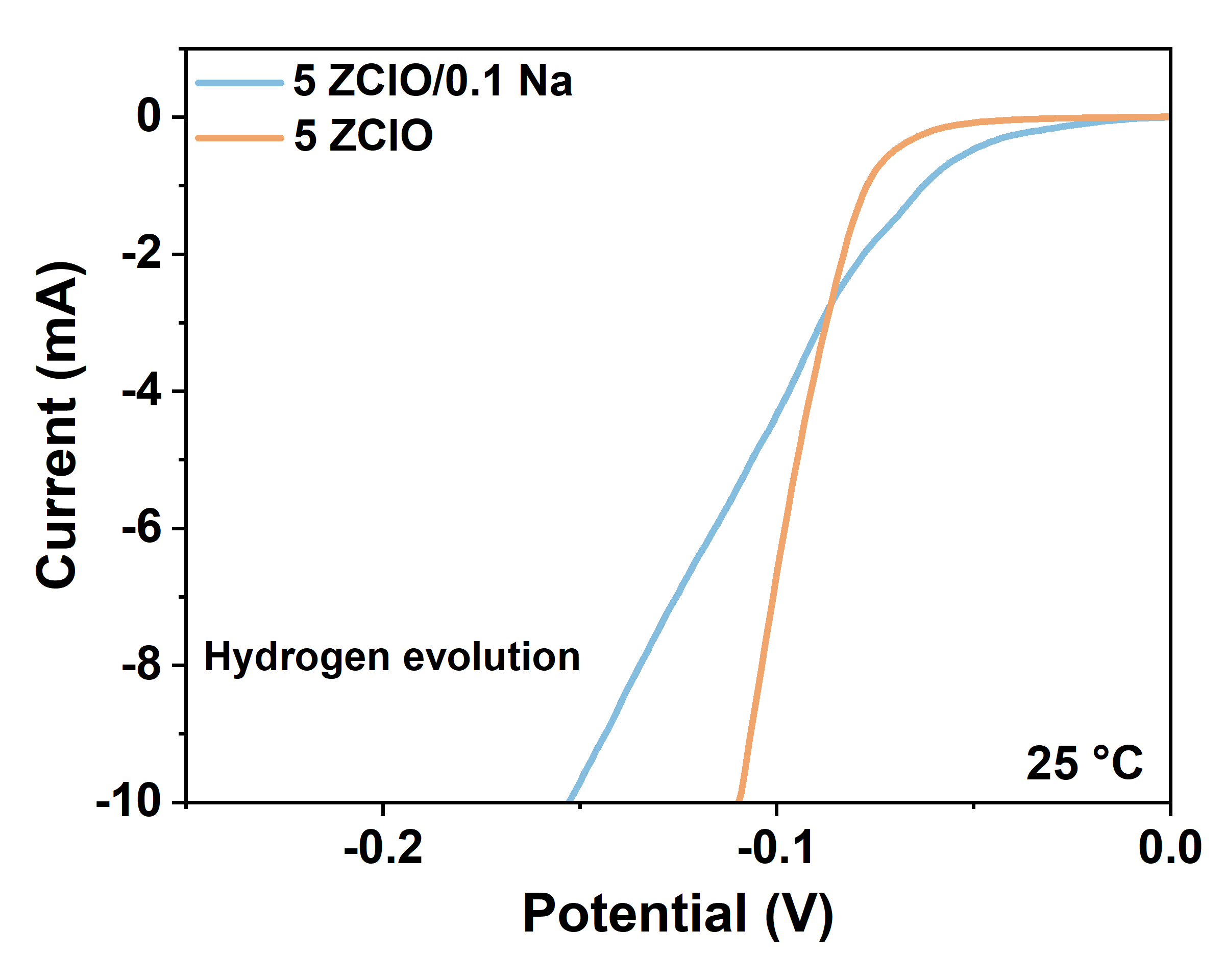


**Fig. S2** LSV curves of 5 ZClO and 5 ZClO/0.1 Na electrolyte


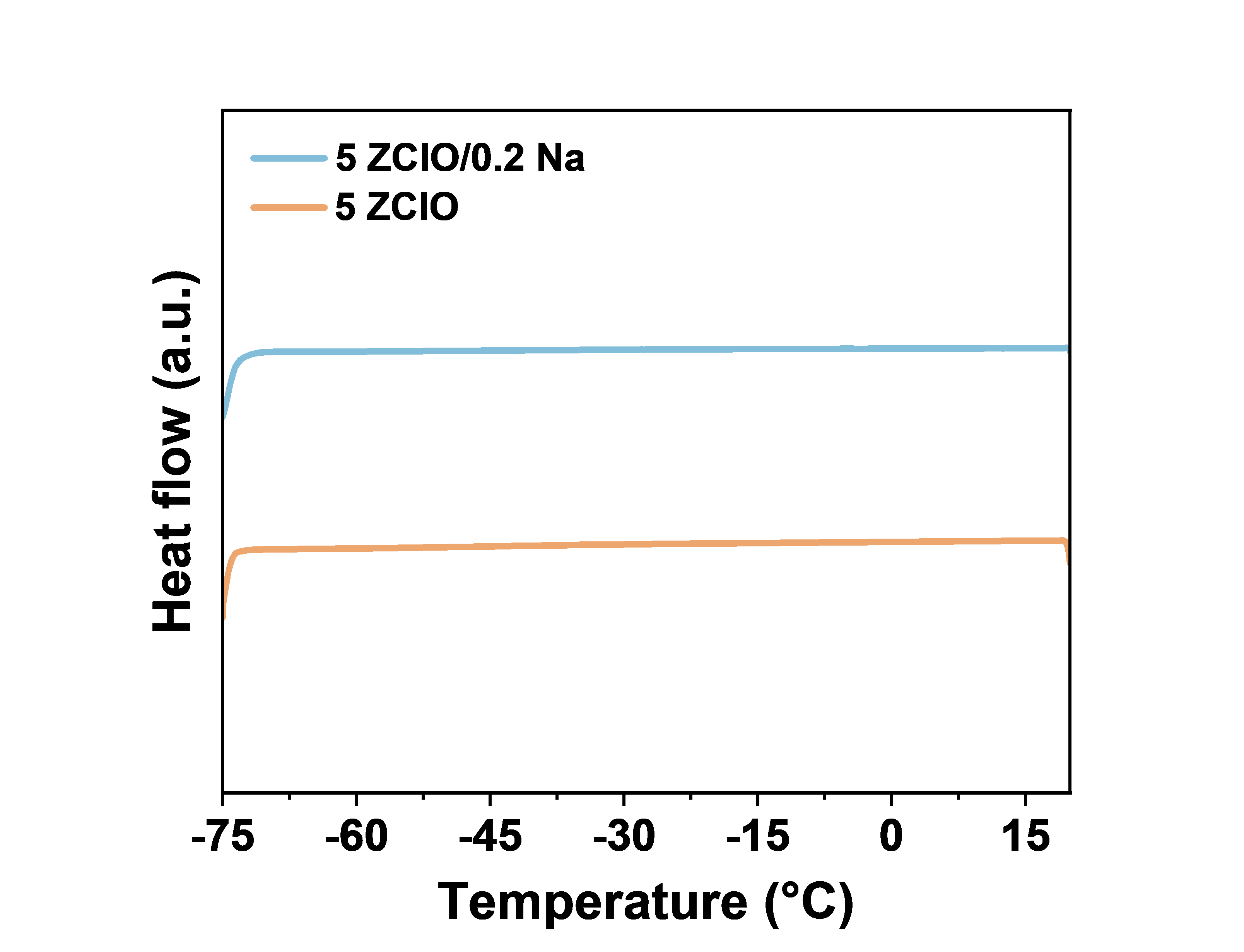


**Fig. S3** DSC test from −75 to 20 ℃ with a heating rate of 10 ℃ min^−1^


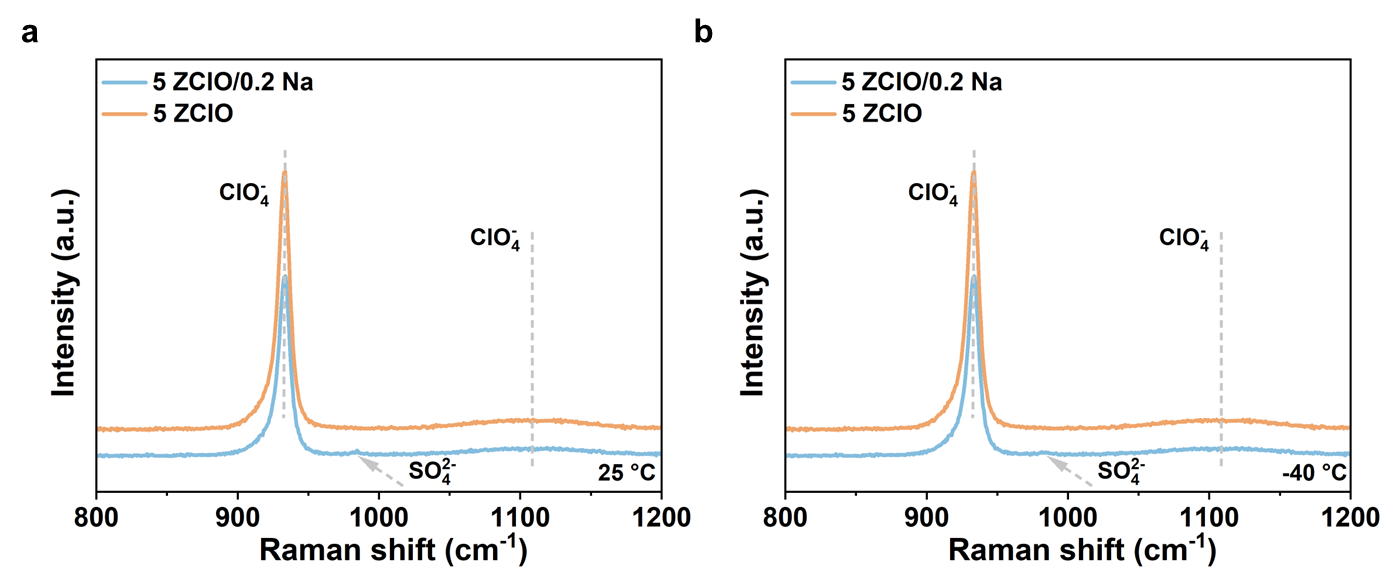


**Fig. S4** Raman spectra of 5 ZClO and 5 ZClO/0.2 Na electrolyte


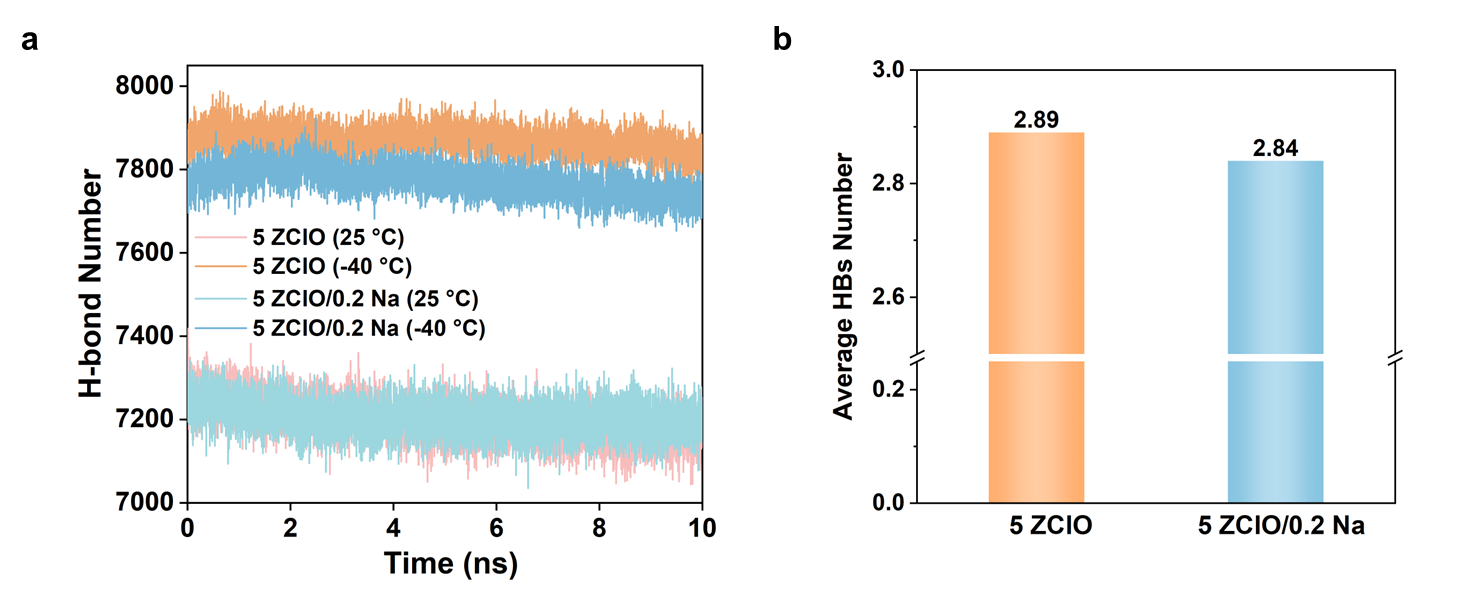


**Fig. S5** (a) The average HBs number in two electrolytes at 25 °C and −40 °C. (b) The average number of HBs formed per water molecule in the electrolyte


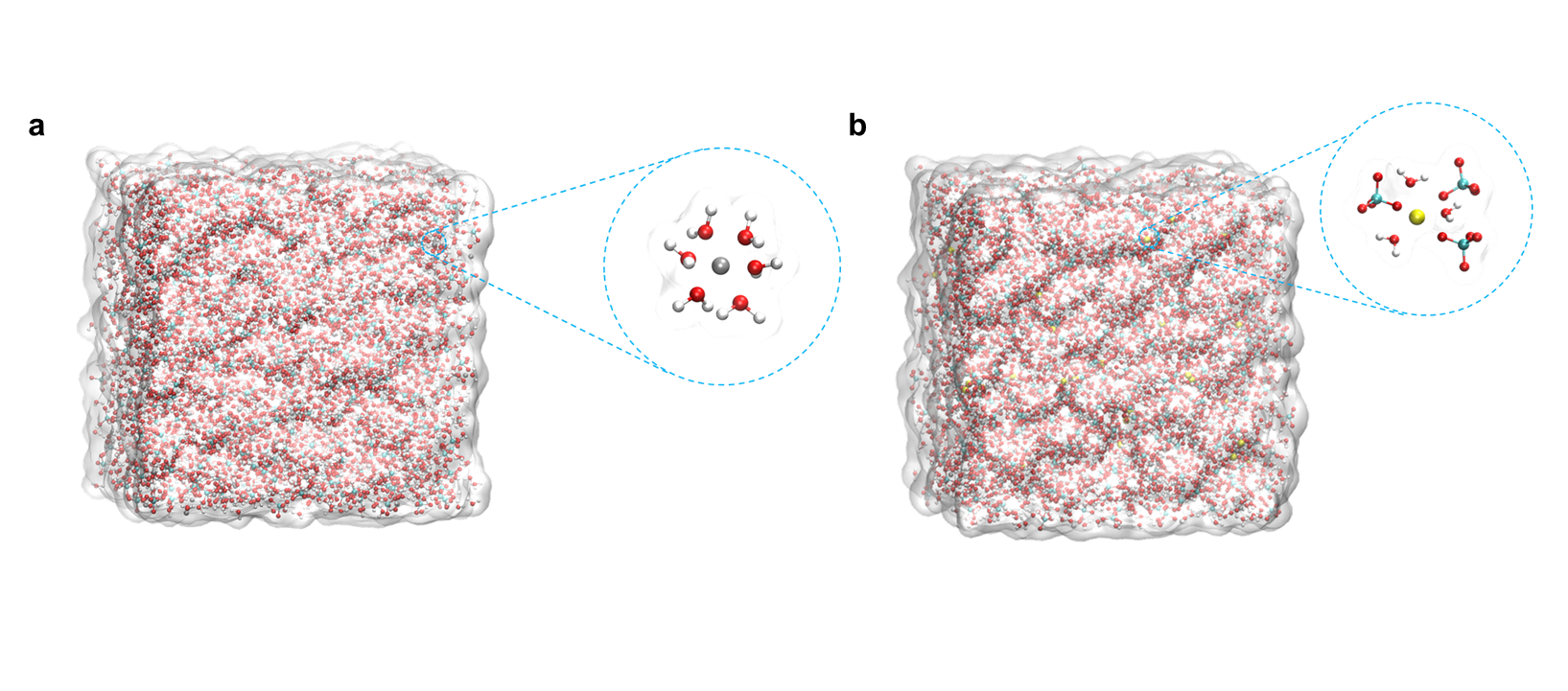


**Fig. S6** Snapshots of the equilibrated MD simulation box for the different electrolytes


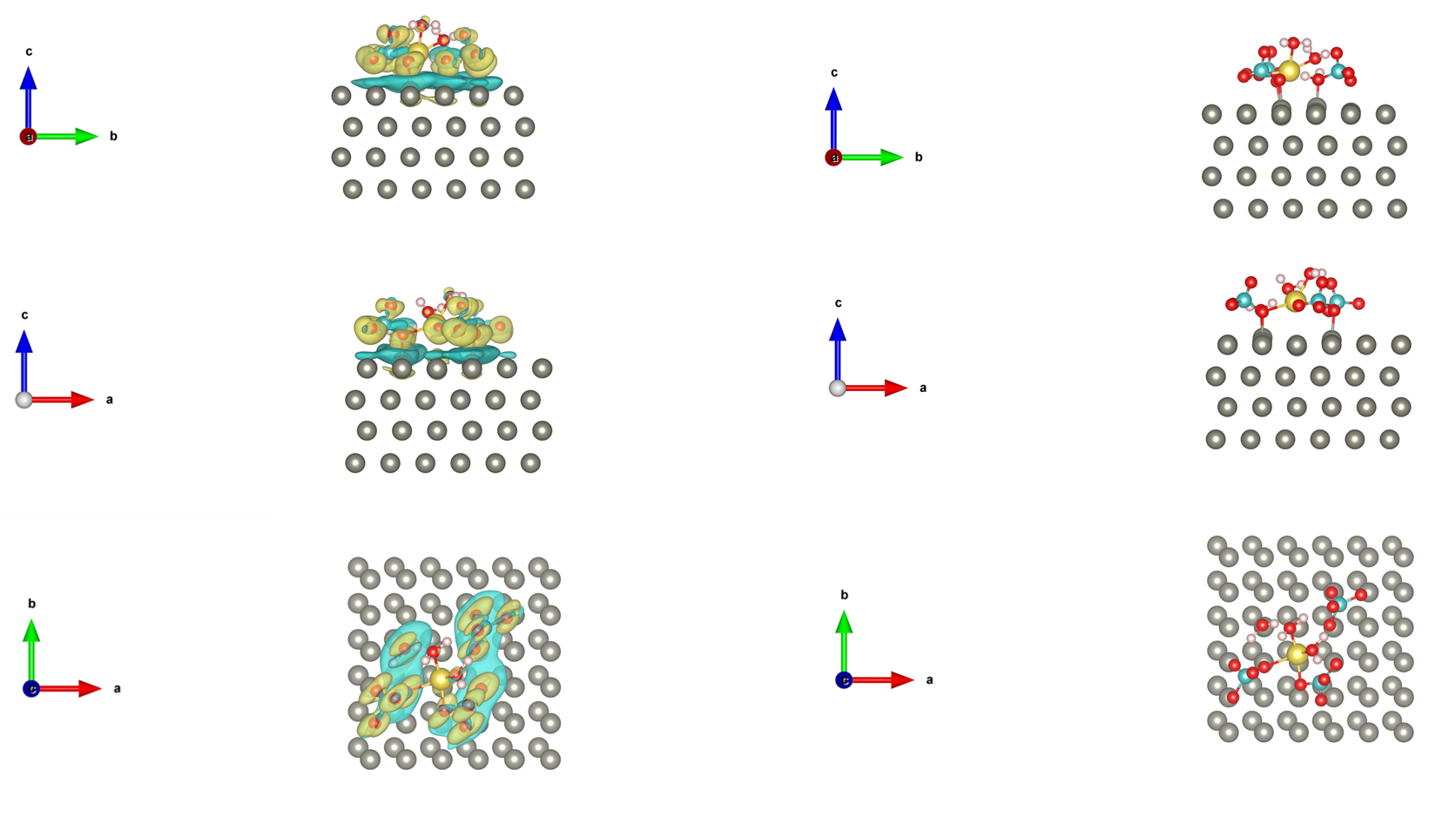


**Fig. S7** The charge density difference for demonstrating interfacial specific adsorption model of anode for Na^+^(H_2_O)3(ClO_4_^−^)_3_. (Cyan: charge depletion; yellow: charge accumulation). The isosurface charge density on the right is removed


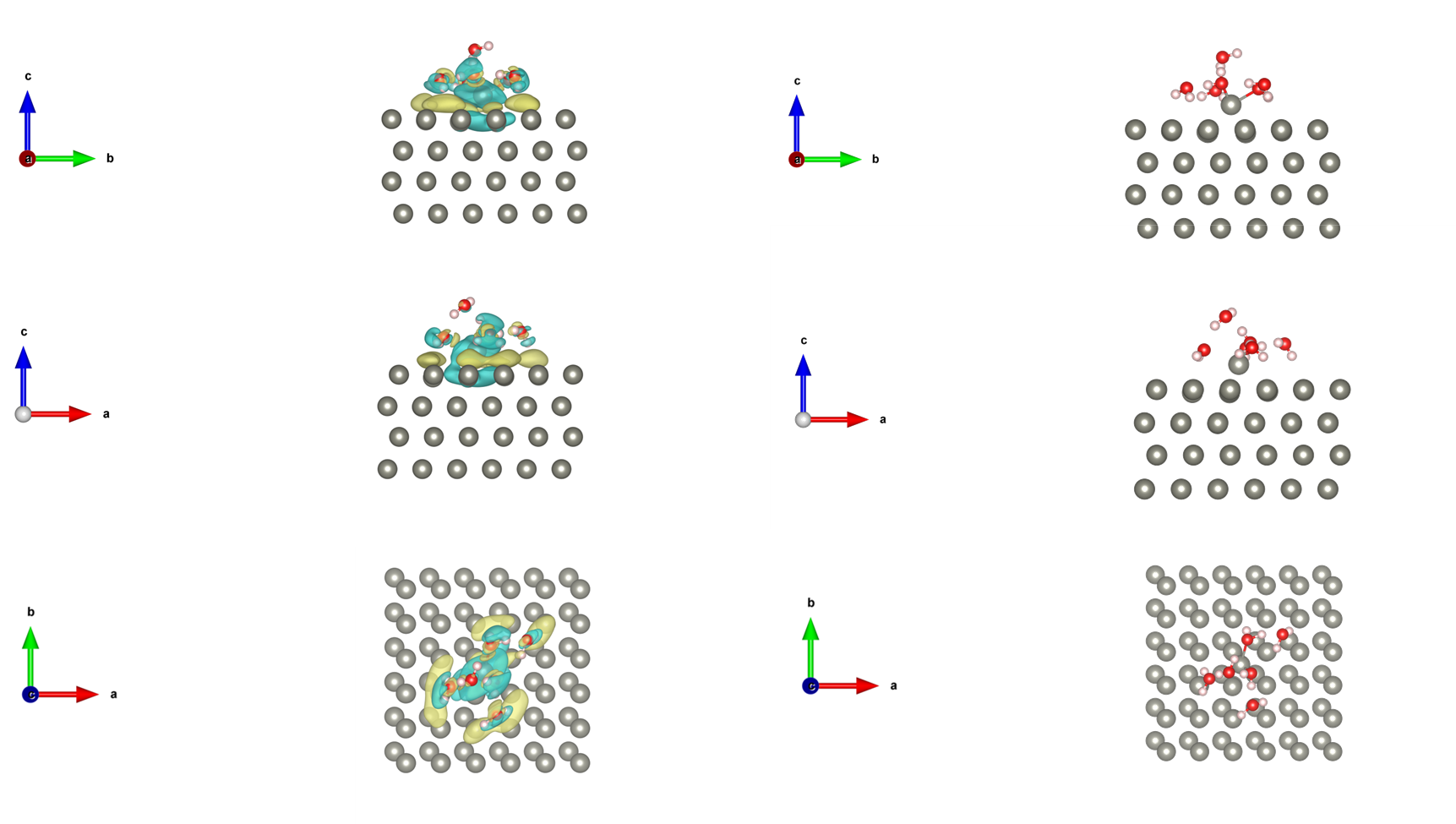


**Fig. S8** The charge density difference for demonstrating interfacial specific adsorption model of anode for Zn^2+^(H_2_O)_6_. (Cyan: charge depletion; yellow: charge accumulation). The isosurface charge density on the right is removed


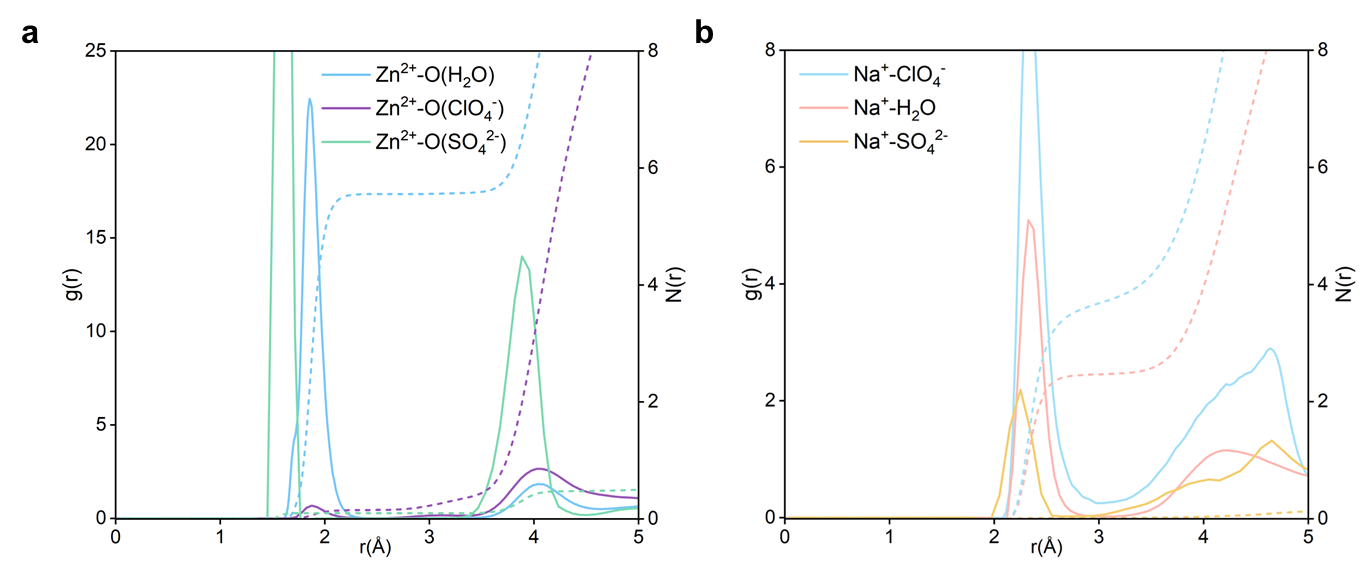


**Fig. S9** RDFs of the primary solvation shell of Zn^2+^ in 5 ZClO and Na^+^ in 5 ZClO/0.2Na at −40 °C


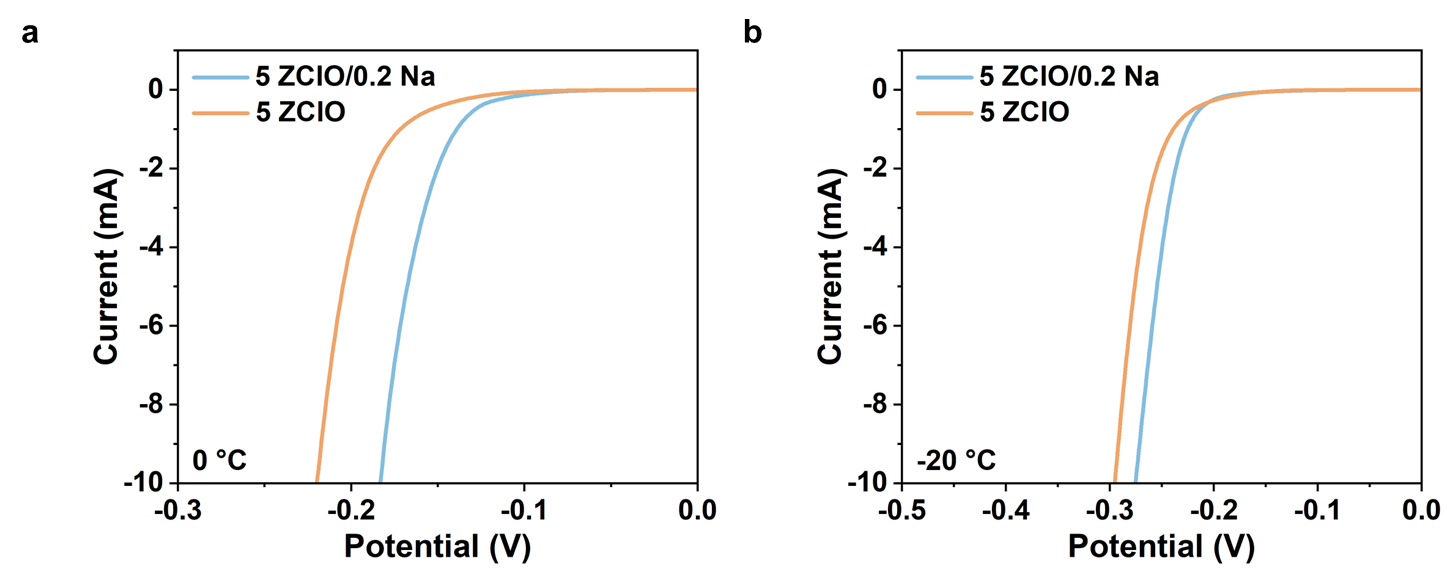


**Fig. S10** LSV curves of 5 ZClO and 5 ZClO/0.2 Na electrolyte at low temperatures


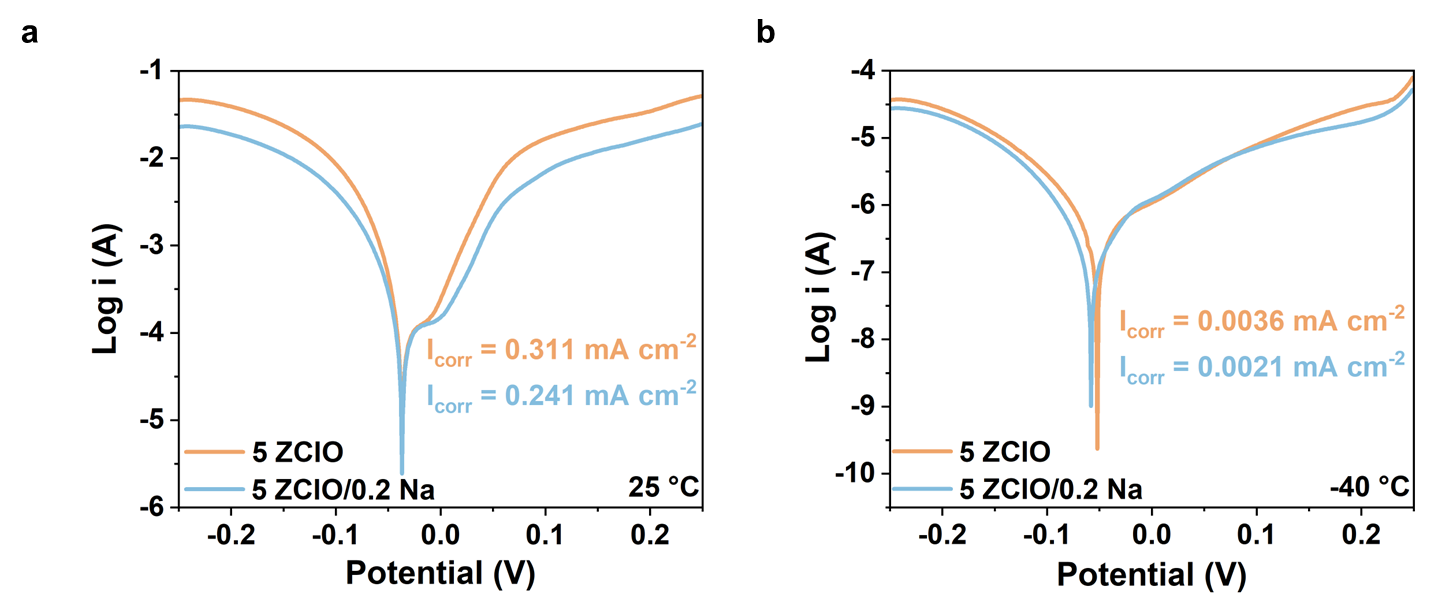


**Fig. S11** Tafel curves of 5 ZClO and 5 ZClO/0.2 Na electrolyte at room temperature low temperature


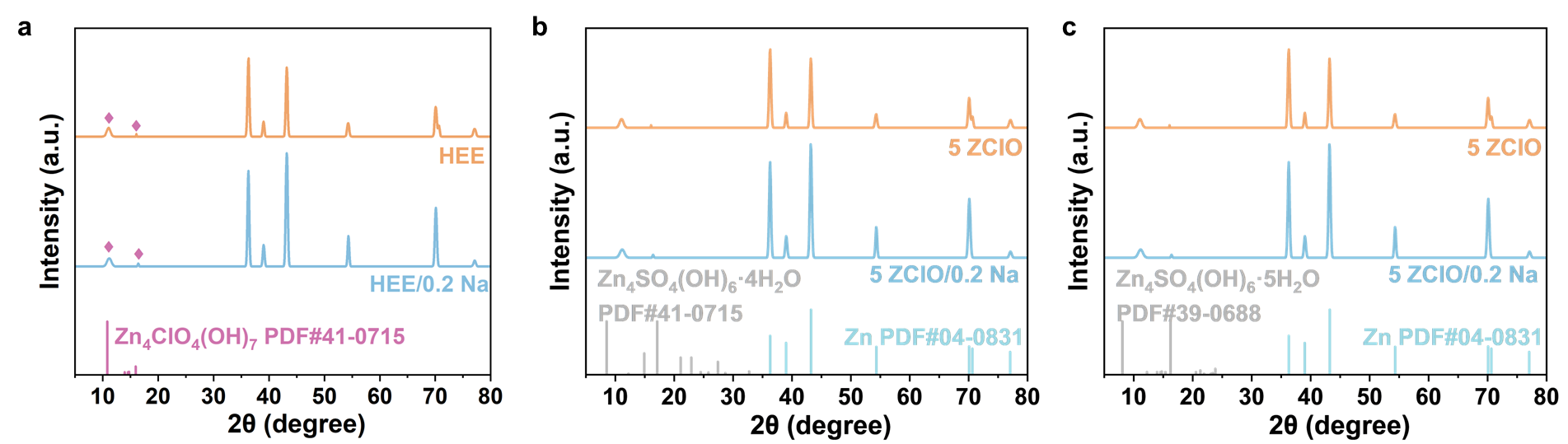


**Fig. S12** The XRD patterns of Zn anodes after cycling 100 cycles at a current density of 5 mA cm^−2^


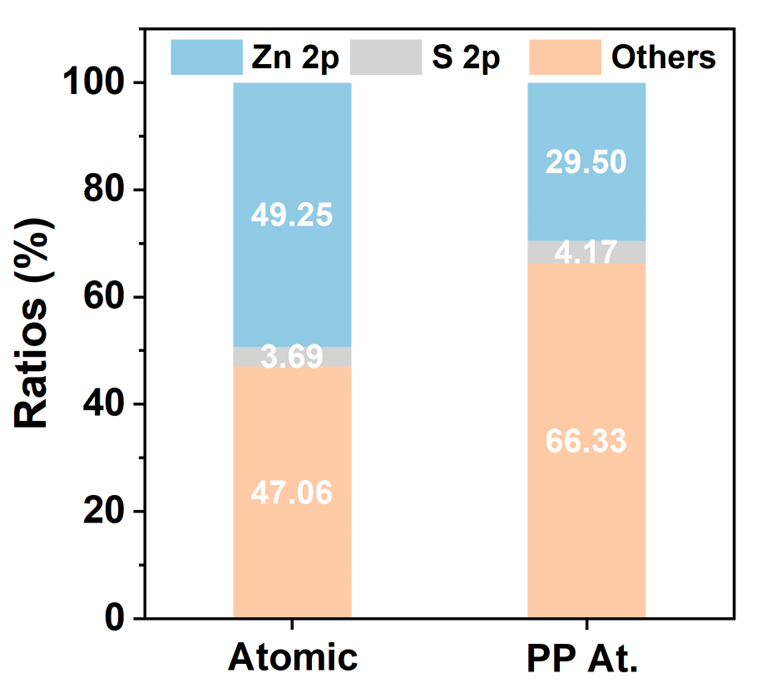


**Fig. S13** The XPS peak area ratio and atomic content ratio of the Zn anodes after cycling 100 cycles at a current density of 5 mA cm^−2^


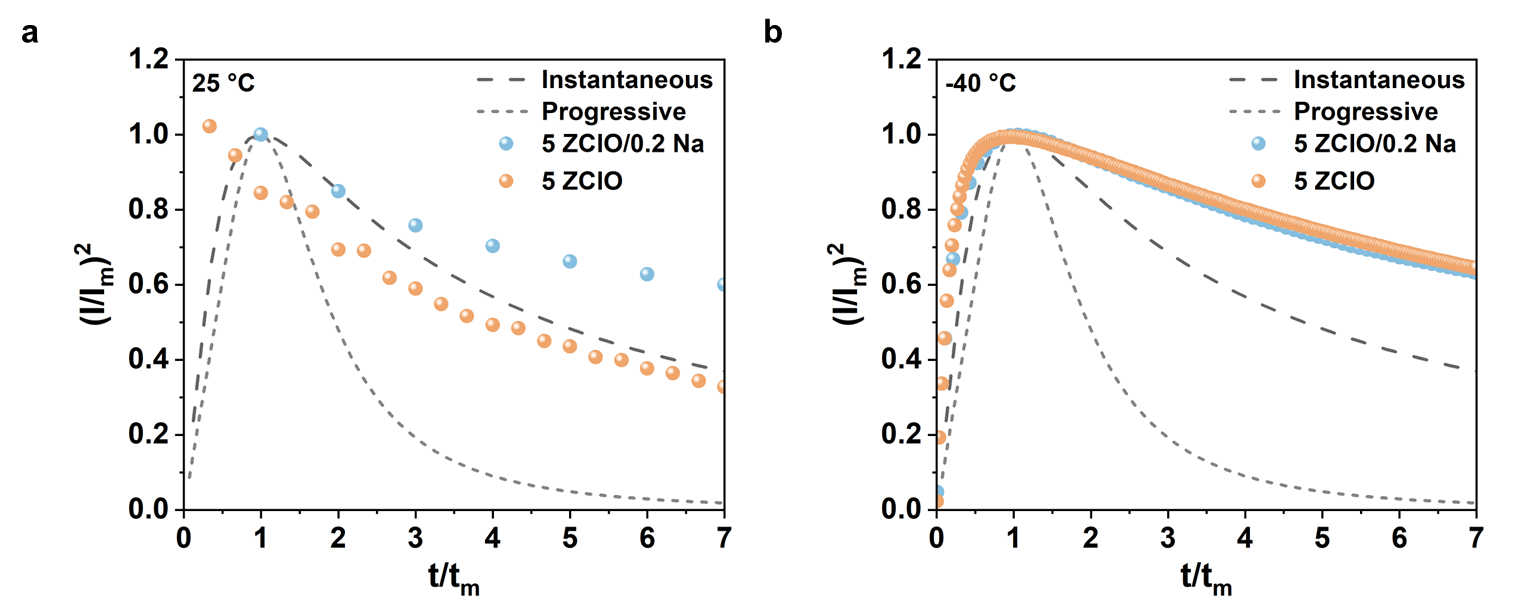


**Fig. S14** (I/I_m_)^2^−(t/t_m_) curves derived from the current response in chronoamperometry plating of Zn at room temperature and low temperature


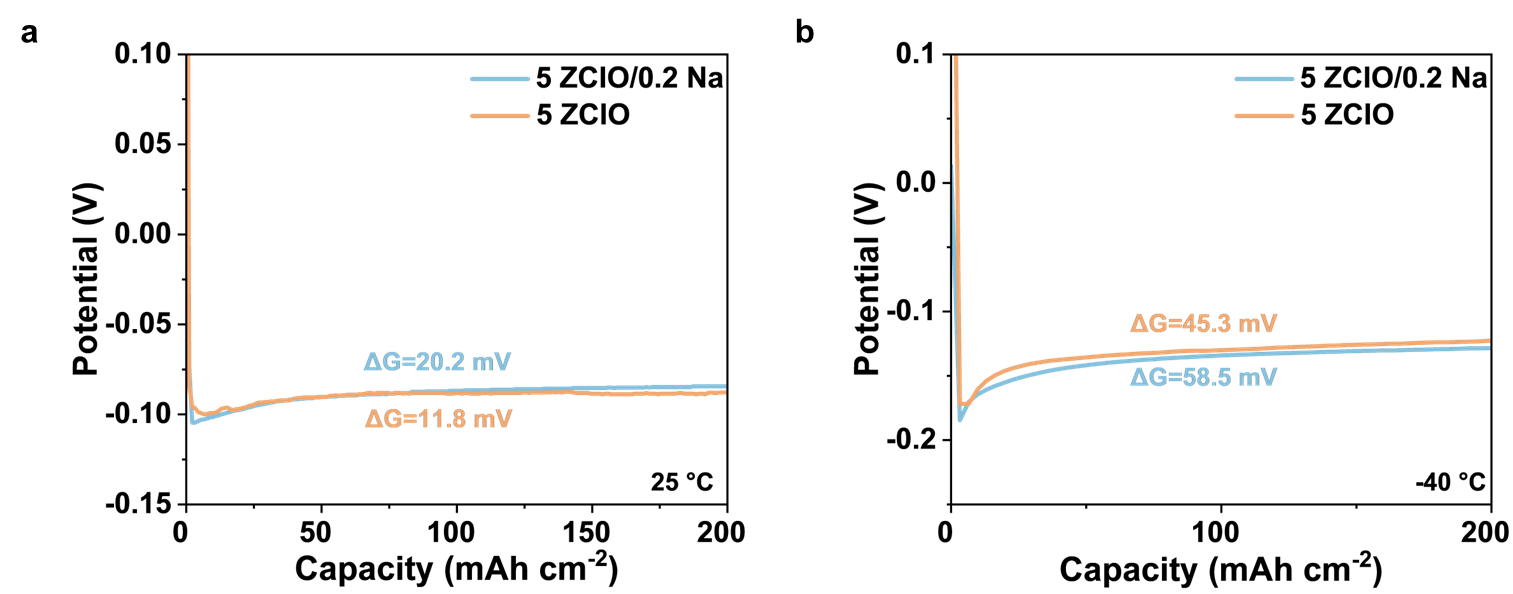


**Fig. S15** Galvanostatic voltage profiles of Zn plating and stripping in different electrolytes


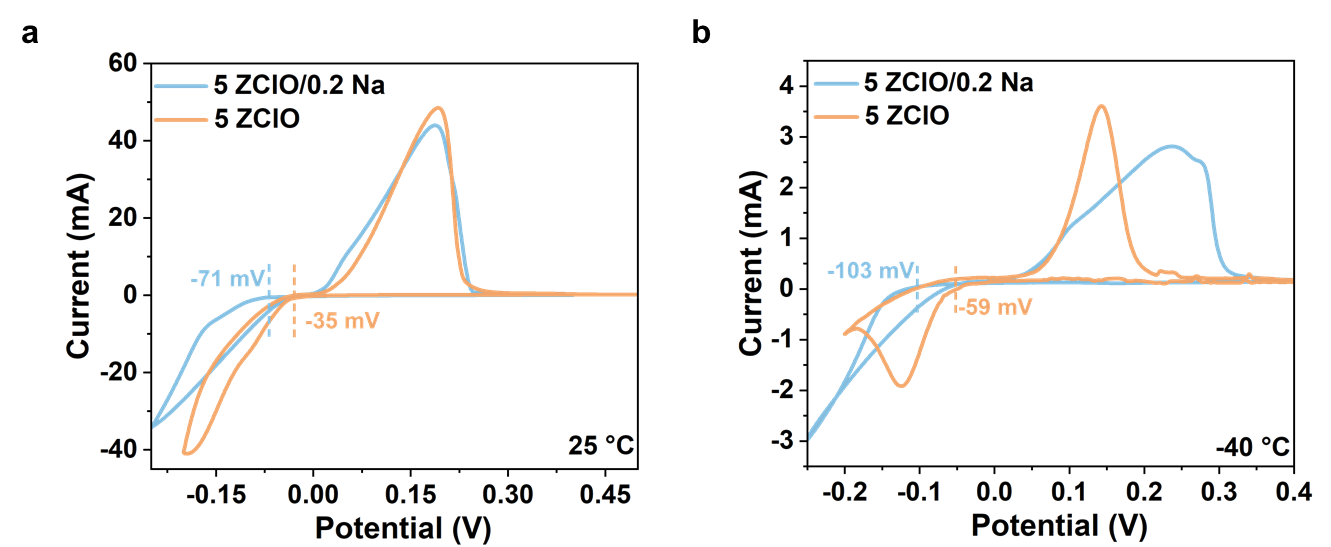


**Fig. S16** Cyclic voltammograms curves of Zn plating and stripping in different electrolytes


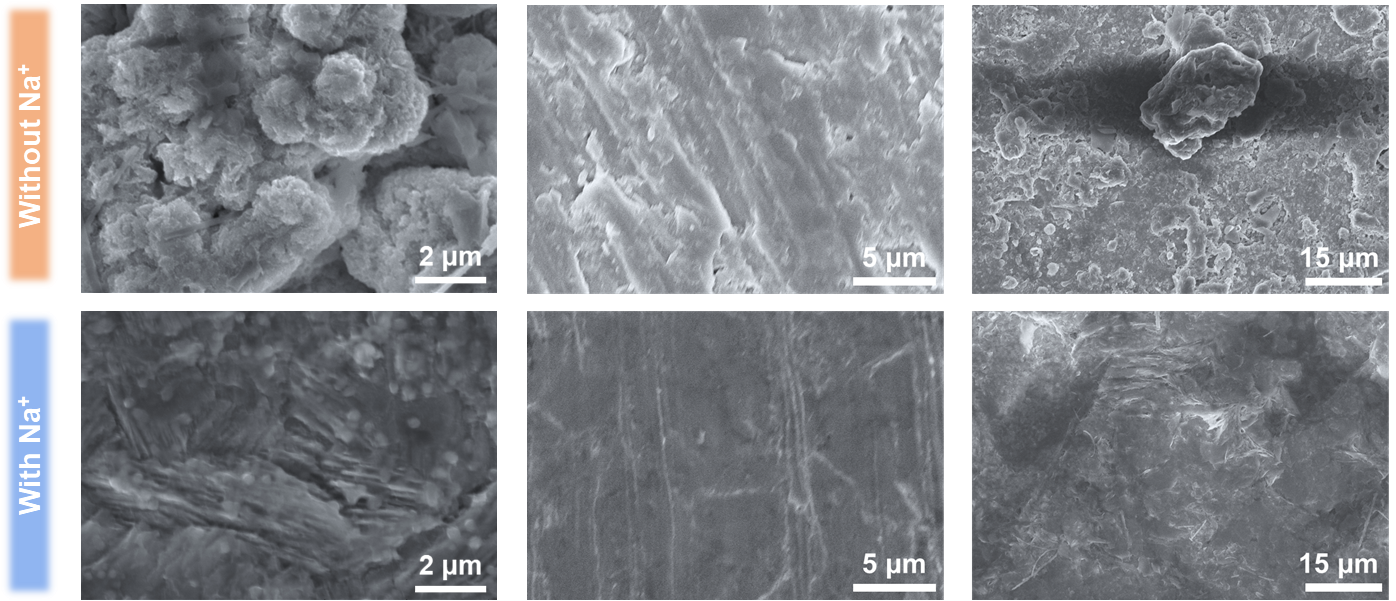


**Fig. S17** SEM image of Zn anodes after cycling 100 cycles at a current density of 5 mA cm^−2^


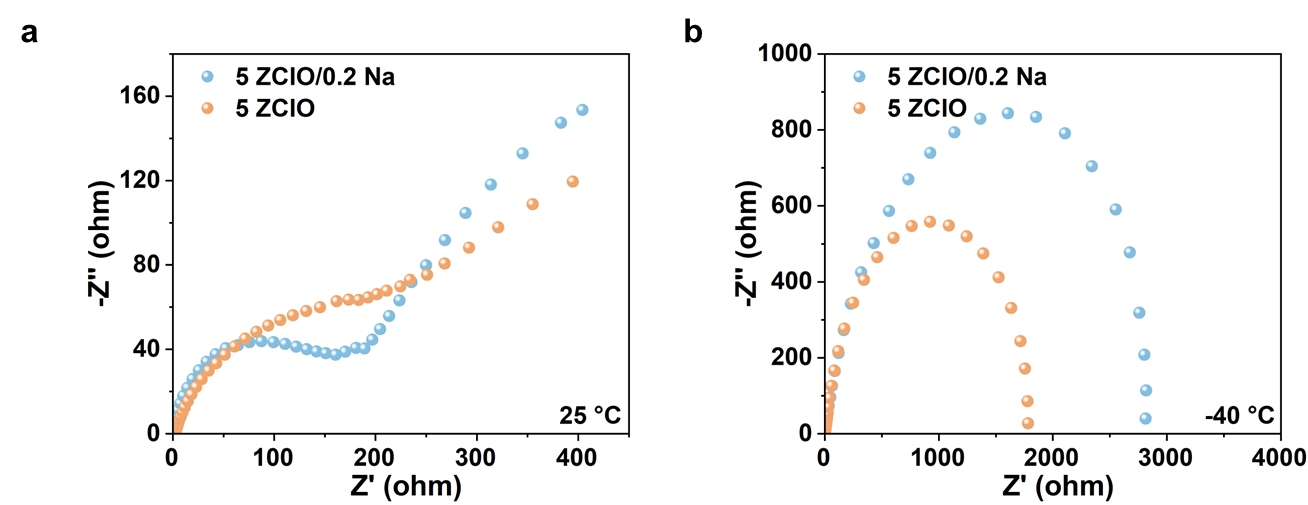


**Fig. S18** Electrochemical impedance spectroscopy (EIS) of the PANI//Zn full battery with different electrolytes


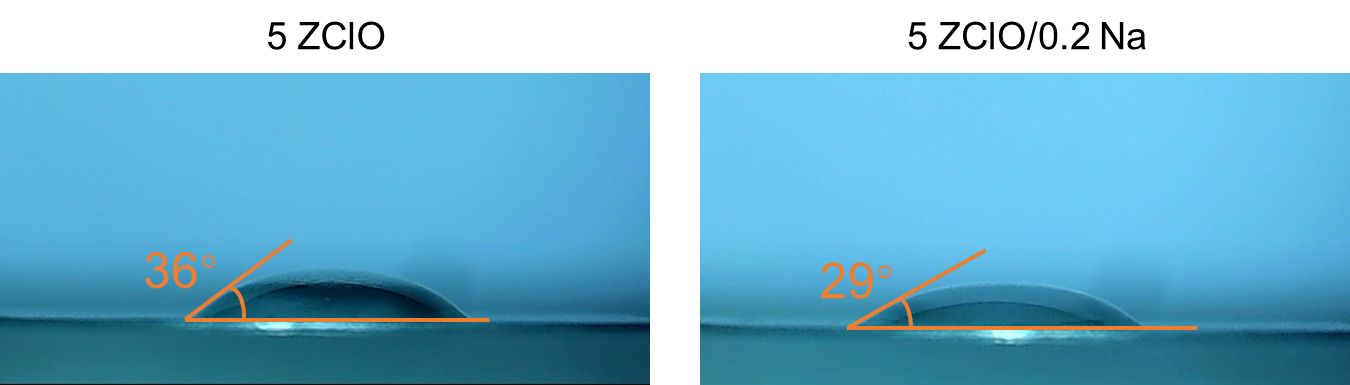


**Fig. S19** Contact angles test of different electrolyte on Zn foil, suggesting their good wettability


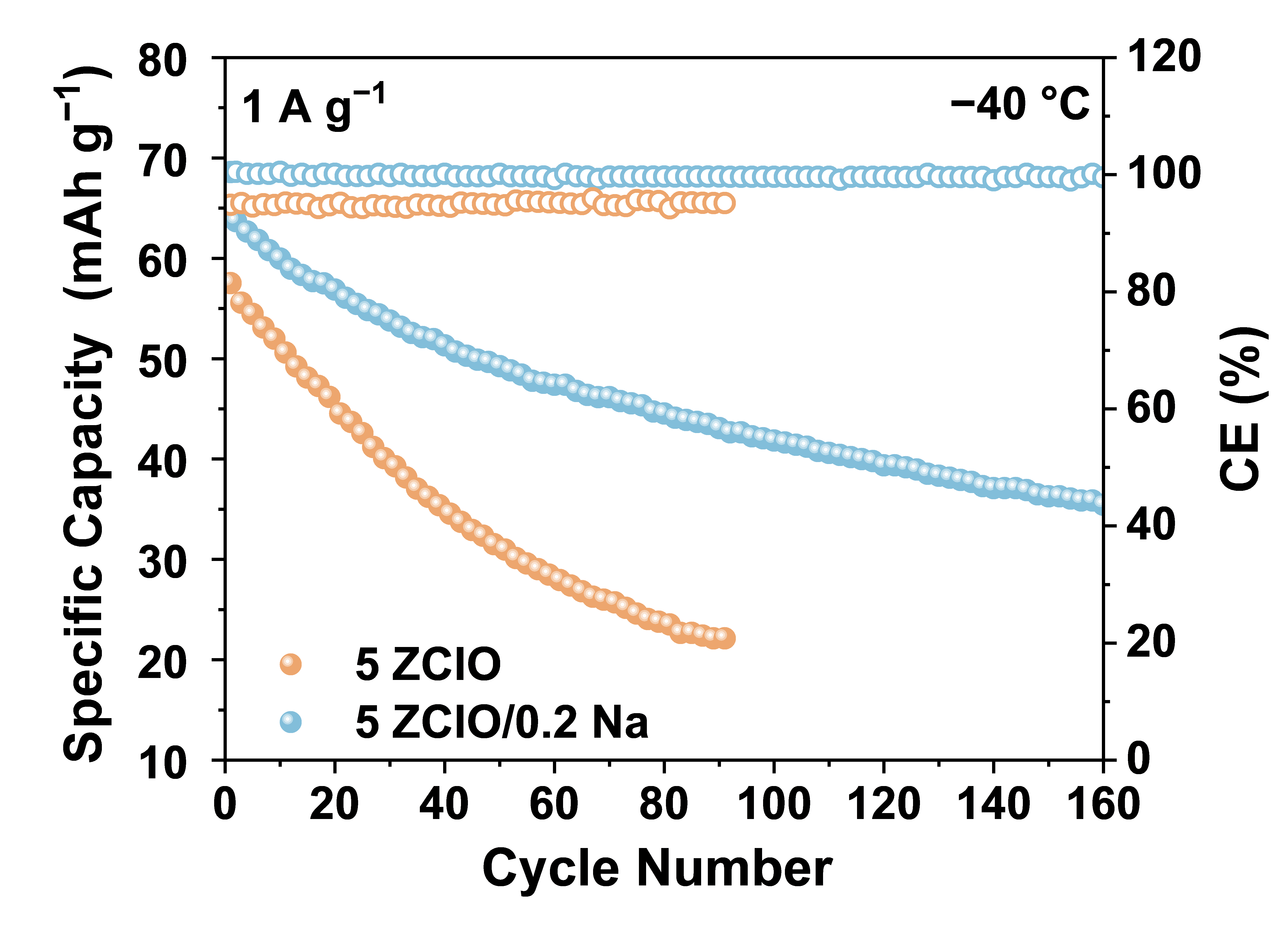


**Fig. S20** The charging/discharging perormancetest of Zn||PANI pouch batteries with different electrolytes at −40°C and a current density of 1A g^−1^


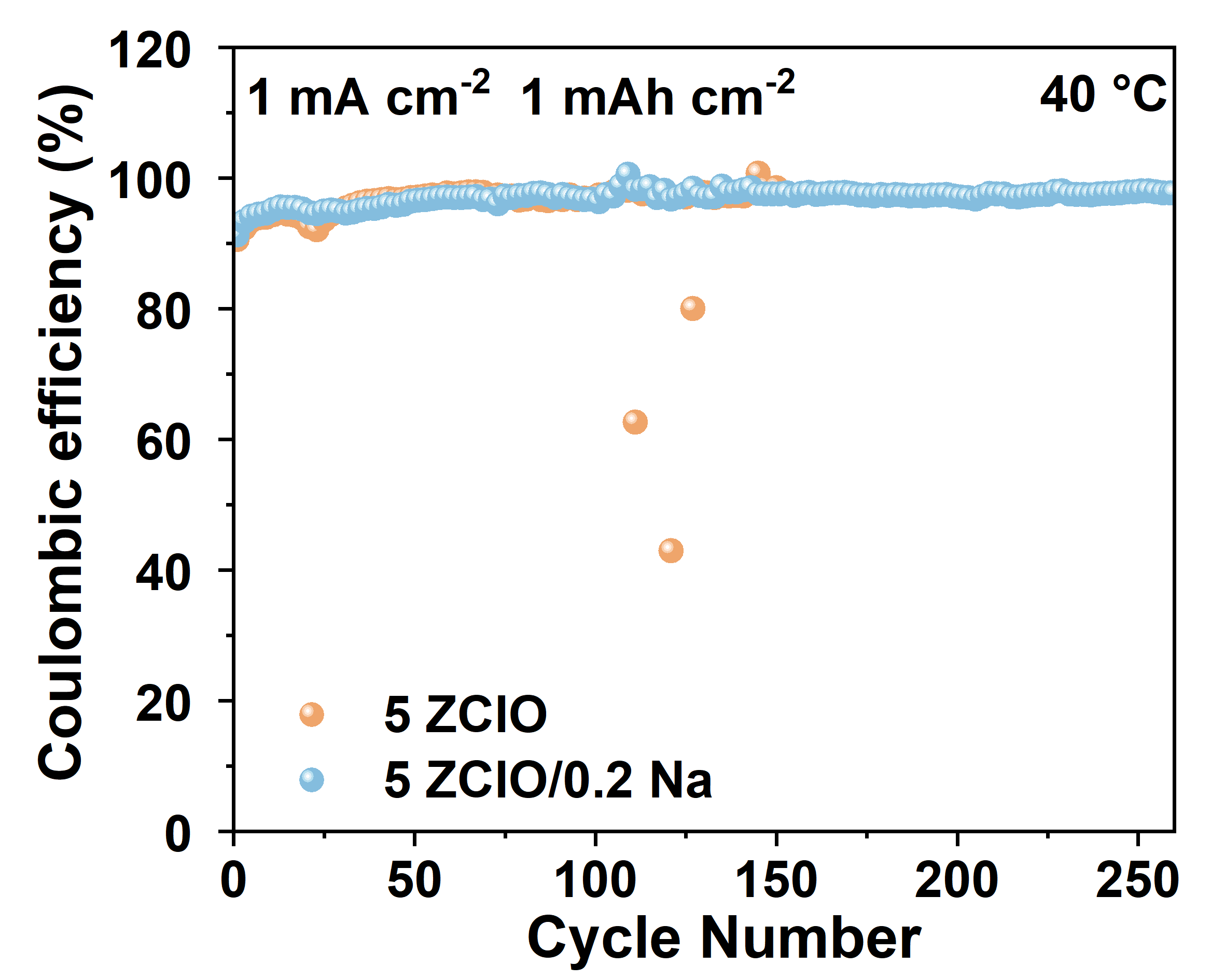


**Fig. S21** Zn plating/stripping CE at 40℃ in different electrolytes

**Supplementary Tables**

**Table S1** The coordination numbers of Zn^2+^ and Na^+^ with H_2_O, ClO_4_^−^, and SO_4_^2−^ in 5 m HEE/0.2 Na electrolyte at 25 °C

|  | H_2_O | ClO_4_^−^ | SO_4_^2−^ |
| --- | --- | --- | --- |
| Zn^2+^ | 5.62 | 0.30 | 0.10 |
| Na^+^ | 2.75 | 3.26 | 0.03 |

**Table S2** The coordination numbers of Zn^2+^ and Na^+^ with H_2_O, ClO_4_^−^, and SO_4_^2−^ in 5 m HEE/0.2 Na electrolyte at −40 °C

|  | H_2_O | ClO_4_^−^ | SO_4_^2−^ |
| --- | --- | --- | --- |
| Zn^2+^ | 5.23 | 0.48 | 0.22 |
| Na^+^ | 3.20 | 2.88 | 0.05 |

**Table S3** The reduction potentials of common metal cations

| Redox Couple | E_q_ (V) |
| --- | --- |
| Li^+^ + e^−^ ⇌ Li | −3.040 |
| Na^+^ + e^−^ ⇌ Na | −2.710 |
| K^+^ + e^−^ ⇌ K | −2.931 |
| Mg^2+^ + 2e^−^ ⇌ Mg | −2.372 |
| Ca^2+^ + 2e^−^ ⇌ Ca | −2.868 |
| Al^3+^ + 3e^−^ ⇌ Al | −1.662 |
| Cu^2+^ + 2e^−^ ⇌ Cu | 0.342 |
| Zn^2+^ + 2e^−^ ⇌ Zn | −0.762 |
| Fe^2+^ + 2e^−^ ⇌ Fe | −0.447 |
| Co^2+^ + 2e^−^ ⇌ Co | −0.280 |
| Ni^2+^ + 2e^−^ ⇌ Ni | −0.257 |

As shown in Table S3, among common metals with large reserves, the standard electrode potentials of Cu, Fe, and Al are too close to that of Zn. They may deposit together during the Zn^2+^ deposition reaction, which is not conducive to providing persistent electrostatic repulsion.
